# Ecophysiology of Freshwater Verrucomicrobia Inferred from Metagenome-Assembled Genomes

**DOI:** 10.1128/mSphere.00277-17

**Published:** 2017-09-27

**Authors:** Shaomei He, Sarah L. R. Stevens, Leong-Keat Chan, Stefan Bertilsson, Tijana Glavina del Rio, Susannah G. Tringe, Rex R. Malmstrom, Katherine D. McMahon

**Affiliations:** a Department of Bacteriology, University of Wisconsin—Madison, Madison, Wisconsin, USA; b Department of Geoscience, University of Wisconsin—Madison, Madison, Wisconsin, USA; c Department of Ecology and Genetics, Limnology and Science for Life Laboratory, Uppsala University, Uppsala, Sweden; d DOE Joint Genome Institute, Walnut Creek, California, USA; e Department of Civil and Environmental Engineering, University of Wisconsin—Madison, Madison, Wisconsin, USA; University of British Columbia

**Keywords:** cytochromes, freshwater, glycoside hydrolase, verrucomicrobia

## Abstract

Microbes are critical in carbon and nutrient cycling in freshwater ecosystems. Members of the *Verrucomicrobia* are ubiquitous in such systems, and yet their roles and ecophysiology are not well understood. In this study, we recovered 19 *Verrucomicrobia* draft genomes by sequencing 184 time-series metagenomes from a eutrophic lake and a humic bog that differ in carbon source and nutrient availabilities. These genomes span four of the seven previously defined *Verrucomicrobia* subdivisions and greatly expand knowledge of the genomic diversity of freshwater *Verrucomicrobia*. Genome analysis revealed their potential role as (poly)saccharide degraders in freshwater, uncovered interesting genomic features for this lifestyle, and suggested their adaptation to nutrient availabilities in their environments. *Verrucomicrobia* populations differ significantly between the two lakes in glycoside hydrolase gene abundance and functional profiles, reflecting the autochthonous and terrestrially derived allochthonous carbon sources of the two ecosystems, respectively. Interestingly, a number of genomes recovered from the bog contained gene clusters that potentially encode a novel porin-multiheme cytochrome *c* complex and might be involved in extracellular electron transfer in the anoxic humus-rich environment. Notably, most epilimnion genomes have large numbers of so-called “*Planctomycete*-specific” cytochrome *c*-encoding genes, which exhibited distribution patterns nearly opposite to those seen with glycoside hydrolase genes, probably associated with the different levels of environmental oxygen availability and carbohydrate complexity between lakes/layers. Overall, the recovered genomes represent a major step toward understanding the role, ecophysiology, and distribution of *Verrucomicrobia* in freshwater.

**IMPORTANCE** Freshwater *Verrucomicrobia* spp. are cosmopolitan in lakes and rivers, and yet their roles and ecophysiology are not well understood, as cultured freshwater *Verrucomicrobia* spp. are restricted to one subdivision of this phylum. Here, we greatly expanded the known genomic diversity of this freshwater lineage by recovering 19 *Verrucomicrobia* draft genomes from 184 metagenomes collected from a eutrophic lake and a humic bog across multiple years. Most of these genomes represent the first freshwater representatives of several *Verrucomicrobia* subdivisions. Genomic analysis revealed *Verrucomicrobia* to be potential (poly)saccharide degraders and suggested their adaptation to carbon sources of different origins in the two contrasting ecosystems. We identified putative extracellular electron transfer genes and so-called “*Planctomycete*-specific” cytochrome *c*-encoding genes and identified their distinct distribution patterns between the lakes/layers. Overall, our analysis greatly advances the understanding of the function, ecophysiology, and distribution of freshwater *Verrucomicrobia*, while highlighting their potential role in freshwater carbon cycling.

## Abstract (precis)

Freshwater *Verrucomicrobia* spp. are cosmopolitan in lakes and rivers, and yet their roles and ecophysiology are not well understood, as cultured freshwater *Verrucomicrobia* spp. are restricted to one subdivision of this phylum. Here, we greatly expanded the known genomic diversity of this freshwater lineage by recovering 19 *Verrucomicrobia* draft genomes from 184 metagenomes collected from a eutrophic lake and a humic bog across multiple years. Most of these genomes represent the first freshwater representatives of several *Verrucomicrobia* subdivisions. Genomic analysis revealed *Verrucomicrobia* to be potential (poly)saccharide degraders and suggested their adaptation to carbon sources of different origins in the two contrasting ecosystems. We identified putative extracellular electron transfer genes and so-called “*Planctomycete*-specific” cytochrome *c*-encoding genes and identified their distinct distribution patterns between the lakes/layers. Overall, our analysis greatly advances the understanding of the function, ecophysiology, and distribution of freshwater *Verrucomicrobia*, while highlighting their potential role in freshwater carbon cycling.

## INTRODUCTION

*Verrucomicrobia* are ubiquitous in freshwater and exhibit a cosmopolitan distribution in lakes and rivers. They are present in up to 90% of lakes (1), with abundances that are typically between <1% and 6% of total microbial communities (2–4) and as high as 19% in a humic lake (5). Yet *Verrucomicrobia* have received less attention than other freshwater bacterial groups, such as members of the *Actinobacteria*, *Cyanobacteria*, and *Proteobacteria* phyla, and their functions and ecophysiology in freshwater are not well understood.

As a phylum, *Verrucomicrobia* was first proposed relatively recently, in 1997 (6). Together with *Planctomycetes*, *Chlamydiae*, and sister phyla such as *Lentisphaerae*, they comprise the *Planctomycetes-Verrucomicrobia-Chlamydiae* (PVC) superphylum. In addition to being cosmopolitan in freshwater, *Verrucomicrobia* have been found in oceans (7, 8), soil (9, 10), wetlands (11), rhizosphere (12), and animal guts (13, 14) as free-living organisms or symbionts of eukaryotes. *Verrucomicrobia* isolates are metabolically diverse and include aerobes, facultative anaerobes, and obligate anaerobes, and they are mostly heterotrophs, using various mono-, oligo-, and polysaccharides for growth (6, 7, 11, 14–20). Not long ago, an autotrophic verrucomicrobial methanotroph (*Methylacidiphilum fumariolicum* SolV) was discovered in acidic thermophilic environments (21).

*Verrucomicrobia* are also ubiquitous in marine environments (22) and have been suggested to have a key role as polysaccharide degraders (23, 24). Genomic insights gained through sequencing single cells (24) or extracting *Verrucomicrobia* bins from metagenomes (25) have revealed high abundances of glycoside hydrolase (GH) genes, providing more evidence for their critical roles in carbon (C) cycling in marine environments.

In freshwater, *Verrucomicrobia* have been suggested to degrade glycolate (26) and polysaccharides (24). The abundance of some phylum members was favored by high nutrient availabilities (27, 28), cyanobacterial blooms (29), low pH, high temperature, high hydraulic retention time (30), and more-labile dissolved organic carbon (DOC) (5). To date, very few freshwater *Verrucomicrobia* have been isolated, including *Verrucomicrobium spinosum* (31) and several *Prosthecobacter* spp. (6). Physiological studies showed that they are aerobes, primarily using carbohydrates but not amino acids, alcohols, or organic acids for growth. However, those few cultured isolates represent only a single clade within subdivision 1. In contrast, 16S rRNA gene-based studies discovered a much wider phylogenic range of freshwater *Verrucomicrobia*, including species representing subdivisions 1, 2, 3, 4, 5, and 6 (3–5, 24, 32). Due to the very few cultured representatives and few available genomes from this freshwater lineage, the ecological functions of the vast uncultured freshwater *Verrucomicrobia* are largely unknown.

In this study, we sequenced a total of 184 metagenomes in a time-series study of two lakes with contrasting characteristics, particularly differing in C sources, nutrient availabilities, and pH. We recovered a total of 19 *Verrucomicrobia* draft genomes spanning subdivisions 1, 2, 3, and 4 of the seven previously defined *Verrucomicrobia* subdivisions. We inferred their metabolisms, revealed their adaptation to C and nutrient conditions, and uncovered some interesting and novel features, including a novel putative porin-multiheme cytochrome *c* (porin-MHC) system that may be involved in extracellular electron transfer (EET). The insights that were gained advanced our understanding of the ecophysiology, potential roles, and ecological niches of this ubiquitous freshwater bacterial group.

## RESULTS AND DISCUSSION

### Comparison of the two lakes.

The two studied lakes exhibited contrasting characteristics (Table 1). The most notable differences are the primary C sources and nutrient availabilities. Mendota is an urban eutrophic lake, with most of its C being autochthonous (produced in-lake through photosynthesis). In contrast, Trout Bog is a nutrient-poor dystrophic lake, surrounded by temperate forests and sphagnum mats and thus receiving large amounts of terrestrially derived allochthonous C that is rich in humic and fulvic acids. Trout Bog features higher DOC levels than Mendota but is more limited in nutrient availability, with much higher DOC/total nitrogen (TN) and DOC/total phosphorus (TP) ratios (Table 1). Nutrient limitation in Trout Bog is even more extreme than is revealed by these ratios because much of the N and P is tied up in complex dissolved organic matter. In addition, Trout Bog has lower levels of oxygenic photosynthesis due to decreased levels of photosynthetically active radiation (PAR) as a result of absorption by DOC (33). Together with the consumption of dissolved oxygen by heterotrophic respiration, oxygen levels decrease quickly with depth in the water column in Trout Bog. Dissolved oxygen is below detectable levels in the hypolimnion nearly year-round (34). Due to these contrasts, we expected to observe differences in bacterial C and nutrient use, as well as differences between these two lakes reflecting the electron acceptor conditions. Hence, the retrieval of numerous *Verrucomicrobia* draft genomes in the two lakes not only allows the prediction of their general functions in freshwater but also provides an opportunity to study their ecophysiological adaptation to the local environmental differences.

**TABLE 1:** Lakes included in this study^a^

Lake parameter	Result	
	Mendota	Trout Bog
GPS location	43.100°N, 89.405°W	46.041°N, 89.686°W
Lake type	Drainage lake	Seepage lake
Surface area (ha)	3,938	1.1
Mean depth (m)	12.8	5.6
Max depth (m)	25.3	7.9
pH	8.3	5.2
Primary carbon source	Phytoplankton	Terrestrial subsidies
DOC (mg/liter)	5.0	20.0
Total N (mg/liter)	1.5	1.3
Total P (μg/liter)	131	71
DOC/N	3.3	15.6
DOC/P	38.0	281.9
Trophic state	Eutrophic	Dystrophic

a Data are from NTL-LTER (https://lter.limnology.wisc.edu) and were averaged from the study years. DOC, dissolved organic carbon; GPS, Global Positioning System; N, nitrogen; P, phosphorus.

### Retrieval of *Verrucomicrobia* draft genomes and their distribution patterns.

A total of 184 metagenomes, including 94 from the top 12 m of Mendota (mostly consisting of the epilimnion layer and therefore referred to here as “ME”), 45 from Trout Bog epilimnion (“TE”), and 45 from Trout Bog hypolimnion (“TH”), were generated from samples collected across multiple years. Three combined assemblies were generated by coassembling reads from all metagenomes within the ME, TE, and TH groups, respectively. Using binning facilitated by tetranucleotide frequency data and relative abundance patterns determined over time, a total of 19 *Verrucomicrobia* metagenome-assembled genomes (MAGs) were obtained, including 8 from the combined assembly of ME, 3 from the combined assembly of TE, and 8 from the combined assembly of TH (Table 2). The 19 MAGs exhibited a clustering of their tetranucleotide frequency data largely based on the two lakes (see Fig. S1 in the supplemental material), suggesting distinct overall genomic signatures associated with each system.

10.1128/mSphere.00277-17.2 FIG S1  A tiled display of an emergent self-organizing map (ESOM) based on the tetranucleotide frequency (TNF) of the 19 *Verrucomicrobia* MAGs. TNF was calculated with a window size of 5 kbp, with each dot on the ESOM representing a 5-kbp fragment (or a contig if its length is shorter than 5 kbp). Dots (i.e., fragments) are colored according to MAGs. A numeric ID is assigned to each MAG; IDs from Mendota are labeled in black, and IDs from Trout Bog labeled in white. A red outline was drawn to indicate the clustering of MAGs from Mendota on the ESOM. Download FIG S1, PDF file, 0.7 MB. Copyright © 2017 He et al. 2017 He et al. This content is distributed under the terms of the Creative Commons Attribution 4.0 International license.

**TABLE 2:** Summary of *Verrucomicrobia* MAGs^a^

Genome	IMG taxon OID	Subdivision	Recovered MAG size (Mbp)^b^	Genome completeness estimate (%)^c^	Genome contamination estimate (%)^c^	GC content (%)	Coding base (%)	Gene count	Normalized coverage depth^d^		
									Median	Mean	Coefficient of variation (%)
**ME3880**	**2582580573**	**1**	**1.6**	**70**	**2**	**58**	**90.9**	**1,585**	**0.2**	**2.9**	**217**
TH2746	2582580664	1	6.5	81	3	62	86.7	5,430	3.3	4.7	82
**ME12612**	**2582580523**	**1**	**2.2**	**79**	**3**	**59**	**89.0**	**2,335**	**0.0**	**0.9**	**261**
**ME12173**	**2582580521**	**1**	**2.1**	**63**	**3**	**52**	**91.3**	**2,070**	**0.0**	**0.8**	**583**
TE4605	2582580638	1	4.7	91	0	59	91.1	4,380	1.0	4.8	198
**ME6381**	**2582580593**	**1**	**2.4**	**62**	**0**	**57**	**92.5**	**2,221**	**0.0**	**0.4**	**285**
**ME8366**	**2582580607**	**2**	**3.6**	**87**	**5**	**63**	**87.4**	**3,450**	**0.0**	**1.2**	**326**
TH2747	2582580665	3	5.2	93	8	58	89.6	4,846	1.8	2.8	99
TH3004	2582580668	3	4.5	93	6	57	91.4	3,798	1.9	5.8	139
TH0989	2556921153	3	7.2	91	8	62	90.3	5,583	6.1	6.3	61
TH2519	2593339181	4	1.8	69	2	42	94.3	1,654	6.2	6.7	60
TE1800	2593339189	4	2.2	84	2	42	94.3	1,998	10.8	11.3	77
TH4590	2582580688	4	3.3	87	1	65	90.7	3,132	2.4	3.6	99
**ME2014**	**2582580546**	**4**	**1.9**	**77**	**5**	**66**	**93.7**	**1,700**	**1.0**	**3.3**	**174**
**ME12657**	**2582580524**	**4**	**1.9**	**81**	**7**	**68**	**94.0**	**1,838**	**0.0**	**0.8**	**344**
TE1301	2582580616	4	2.0	95	0	54	94.7	1,943	4.1	14.2	187
TH4093	2582580682	Unclassified	4.7	77	6	48	86.5	3,982	4.3	3.9	61
**ME30509**	**2582580559**	**Unclassified**	**1.2**	**51**	**2**	**63**	**92.3**	**1,160**	**0.0**	**0.5**	**479**
TH4820	2582580691	Unclassified	3.0	56	3	63	86.7	2,794	1.0	2.1	110

a MAGs from Lake Mendota are indicated in boldface.

b Recovered MAG size data represent the sum of the length of all contigs within a MAG.

c Genome completeness and contamination were estimated with checkM using *Verrucomicrobia*-specific marker gene sets.

d Normalized coverage depths of MAGs were calculated from the 94, 45, or 45 individual ME, TE, or TH metagenomes, respectively, and were used to comparatively infer relative population abundances at the different sampling points. In addition to the median and mean coverage depths, the coefficient of variation is also shown to indicate variation among the sampling points.

**FIG 1:**
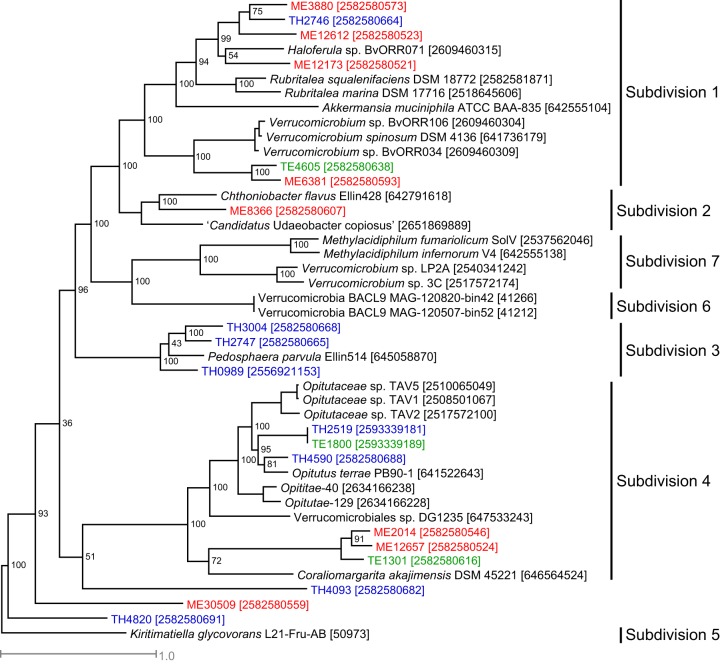
Phylogenetic tree constructed with a concatenated alignment of protein sequences from five conserved essential single-copy genes (represented by TIGR01391, TIGR01011, TIGR00663, TIGR00460, and TIGR00362) that were recovered in all *Verrucomicrobia* MAGs. ME, TE, and TH MAGs are labeled with red, green, and blue, respectively. Genome ID in IMG or NCBI is indicated in the bracket. The outgroup is *Kiritimatiella glycovorans* L21-Fru-AB, which was initially assigned to subdivision 5, but this subdivision was recently proposed as a novel sister phylum to *Verrucomicrobia* (67).

Genome completeness of the 19 MAGs ranged from 51% to 95%, as determined by checkM (35). Phylogenetic analysis of these MAGs using a concatenated alignment of their conserved genes indicates that they span a wide phylogenetic spectrum and distribute in subdivisions 1, 2, 3, and 4 of the seven previously defined *Verrucomicrobia* subdivisions (5, 21, 36) (Fig. 1), as well as three unclassified *Verrucomicrobia* MAGs.

Presently available freshwater *Verrucomicrobia* isolates are restricted to subdivision 1. The recovered MAGs allow the inference of metabolisms and ecology of a considerable diversity within uncultured freshwater *Verrucomicrobia*. Notably, all MAGs from subdivision 3 were recovered from TH, and all MAGs from subdivision 1, except TH2746, were from the epilimnion (either ME or TE), indicating differences in phylogenetic distributions between lakes and between layers within a lake.

We used normalized coverage depths of MAGs within individual metagenomes collected at different sampling time points and in different lakes/layers to comparatively infer relative population abundances across time and space (see detailed coverage depth estimations in Text S1 in the supplemental material). Briefly, we mapped reads from each metagenome to MAGs with a minimum identity of 95% and used the number of mapped reads to calculate the relative abundance for each MAG based on coverage depth per contig and several normalization steps. Thus, we assumed that each MAG represents a distinct population within the lake layer from which it was recovered (37, 38). This estimate does not directly indicate the actual relative abundances of these populations within the total community *per se*; rather, it allows us to compare the levels of abundance of populations from different lakes and sampling occasions within the set of 19 MAGs. This analysis indicates that *Verrucomicrobia* populations in Trout Bog were proportionally more abundant and persistent over time than those in Mendota in general (Table 2). *Verrucomicrobia* populations in Mendota boosted their abundances once to a few times during the sampling season and diminished to extremely low levels for the remainder of the sampling season (generally May to November), as reflected by the low median coverage depth of Mendota MAGs and their large coefficient of variation (Table 2).

10.1128/mSphere.00277-17.1 TEXT S1  Supplemental text. Download TEXT S1, DOCX file, 0.2 MB. Copyright © 2017 He et al. 2017 He et al. This content is distributed under the terms of the Creative Commons Attribution 4.0 International license.

### Saccharolytic lifestyle and adaptation to different C sources.

*Verrucomicrobia* isolates from different environments are known to grow on various mono-, oligo-, and polysaccharides but are unable to grow on amino acids, alcohols, or most organic acids (6, 7, 11, 14–20, 39). Culture-independent research suggests that marine *Verrucomicrobia* are candidate polysaccharide degraders with large numbers of genes involved in polysaccharide utilization (23–25).

**FIG 2:**
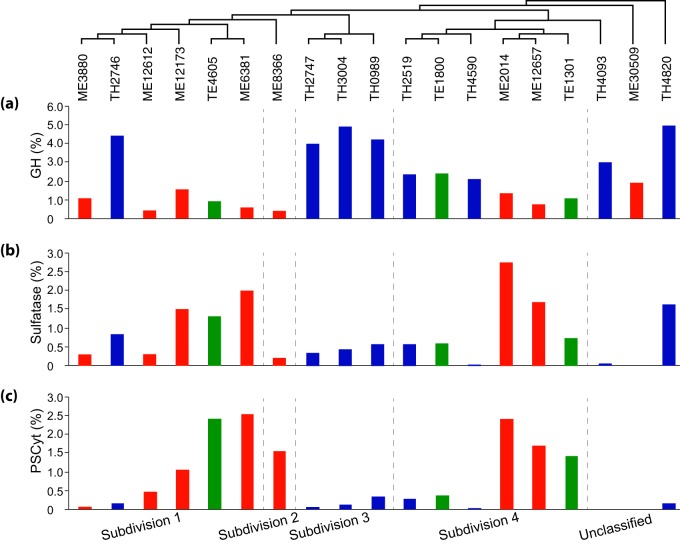
Coding densities of (a) glycoside hydrolase genes, (b) sulfatase genes, and (c) *Planctomycete*-specific cytochrome *c* (PSCyt)-encoding genes. Data from ME, TE, and TH MAGs are labeled with red, green, and blue, respectively. The three plots share the same *x* axis label as indicated by the genome clustering on the top, which is based on a subtree extracted from the phylogenetic tree in Fig. 1 to indicate the phylogenetic relatedness of the 19 MAGs. The vertical dashed lines divide these MAGs to different subdivisions.

**FIG 3:**
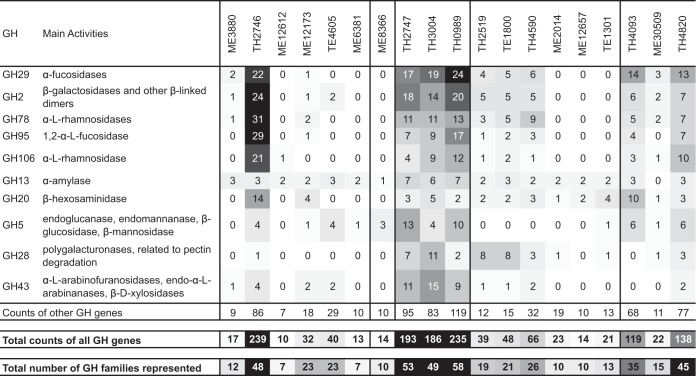
Gene counts for the top 10 most abundant GH families, total gene counts for all GH families, and the number of GH families represented by these genes. MAGs are ordered as in the clustering in Fig. 2. Data were shaded in grayscale according to the value, with the largest value shaded with the darkest color (black) and the smallest value with the brightest color (white) to highlight the difference. The shading scale was applied among the counts of the top 10 GHs, as well as among total counts of all GHs and among total number of GH families.

In the 19 *Verrucomicrobia* MAGs, we observed rich arrays of GH genes, representing a total of 78 different GH families acting on diverse polysaccharides (Fig. S2). Although these genomes have different degrees of completeness, genome completeness was not correlated with the number of GH genes recovered (correlation coefficient = 0.312, *P* value = 0.194) or with the number of GH families represented in each MAG (i.e., GH diversity; correlation coefficient = 0.278, *P* value = 0.250). To compare GH abundance among MAGs, we normalized GH occurrence frequencies by the total number of genes in each MAG to estimate the percentage of genes annotated as GHs (i.e., GH coding density) to account for the different genome sizes and completeness levels. This normalization method assumes that GH genes are randomly distributed between the recovered and the missing parts of the genome, and it allows us to make some general comparisons among these MAGs. GH coding density ranged from 0.4% to 4.9% for these MAGs (Fig. 2a) and, in general, was higher in Trout Bog MAGs than in Mendota MAGs. Notably, six TH MAGs had extremely high (~4%) GH coding densities (Fig. 2a), with each MAG harboring 119 to 239 GH genes, representing 36 to 59 different GH families (Fig. 3 and Fig. S2). Although the GH coding density in most ME genomes in subdivisions 1 and 2 was relatively low (0.4 to 1.6%), it was still higher than in many other bacterial groups (24).

10.1128/mSphere.00277-17.3 FIG S2  Counts of GH genes among the 78 different GH families present in MAGs. Download FIG S2, PDF file, 0.1 MB. Copyright © 2017 He et al. 2017 He et al. This content is distributed under the terms of the Creative Commons Attribution 4.0 International license.

The GH abundance and diversity within a genome may determine the width of the substrate spectrum and/or the complexity of the carbohydrates used by that organism. For example, there are 20 GH genes in the *Rubritalea marina* genome, and this marine verrucomicrobial aerobe uses only a limited spectrum of carbohydrate monomers and dimers but not the majority of (poly)saccharides tested (15). In contrast, 164 GH genes are present in the *Opitutus terrae* genome, and this soil verrucomicrobial anaerobe can thus grow on a wider range of mono-, di-, and polysaccharides (16). Therefore, it is plausible that the GH-rich Trout Bog *Verrucomicrobia* populations may be able to use a wider range of more-complex polysaccharides than the Mendota populations.

The 10 most abundant GH families in these *Verrucomicrobia* MAGs include GH2, GH29, GH78, GH95, and GH106 (Fig. 3). These specific GHs were absent or were present at very low abundances in marine *Verrucomicrobia* genomes (24, 25), suggesting a general difference in carbohydrate substrate use between freshwater and marine *Verrucomicrobia*. Hierarchical clustering of MAGs based on overall GH abundance profiles indicated grouping patterns that were largely separated by lake (Fig. S3). Prominently overrepresented GHs in most Trout Bog MAGs include GH2, GH29, GH78, GH95, and GH106. In contrast, overrepresented GHs in the Mendota MAGs are GH13, GH20, GH33, GH57, and GH77, which have substrate spectra that differ from those of the GHs overrepresented in the Trout Bog MAGs. Therefore, the patterns in GH functional profiles may suggest different carbohydrate substrate preferences and ecological niches occupied by *Verrucomicrobia*, probably reflecting the different carbohydrate compositions derived from different sources between Mendota and Trout Bog.

10.1128/mSphere.00277-17.4 FIG S3  Heat map based on GH abundance profile patterns showing the clustering of MAGs by different lakes. Download FIG S3, PDF file, 0.6 MB. Copyright © 2017 He et al. 2017 He et al. This content is distributed under the terms of the Creative Commons Attribution 4.0 International license.

Overall, GH diversity and abundance profile may reflect the DOC availability and chemical variety and complexity and may suggest microbial adaptation to different C sources in the two ecosystems. We speculate that the rich arrays of GH genes and presumably broader substrate spectra of Trout Bog populations partly contribute to their higher abundance and persistence over the sampling season (Table 2), as they are less likely impacted by fluctuations of individual carbohydrates. In contrast, Mendota populations with fewer GHs and presumably more-specific substrate spectra may rely on autochthonous C and therefore exhibit a “bloom-and-bust” abundance pattern (Table 2) that might be associated with cyanobacterial blooms as previously suggested (29). On the other hand, bogs experience seasonal phytoplankton blooms (40, 41) that introduce brief pulses of autochthonous C to these otherwise allochthonously driven systems. Clearly, much remains to be learned about the routes through which C is metabolized by bacteria in such lakes, and comparative genomics is a novel way to use the organisms to tell us about C flow through the ecosystem.

### Other genome features of the saccharide-degrading lifestyle.

**FIG 4:**
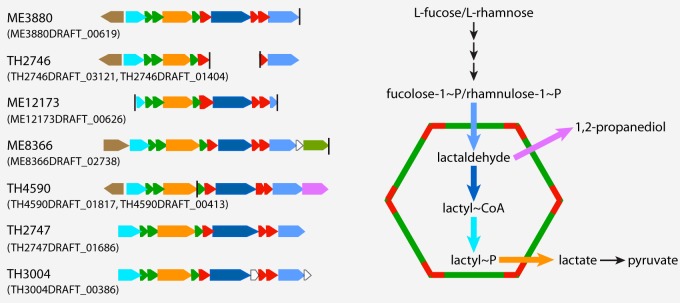
Gene clusters encoding bacterial microcompartments (BMCs) involved in l-fucose and l-rhamnose degradation. The vertical line indicates the end of a contig, and IMG gene locus tag for the first gene in each presented gene cluster is indicated in parentheses. The BMC is schematically represented by a hexagon with the two building blocks labeled in red and green, respectively. The two building blocks and reactions inside the BMC are indicated with colored labels corresponding to the colors used with the encoding genes on the left side.

Seven *Verrucomicrobia* MAGs spanning subdivisions 1, 2, 3, and 4 possess genes needed to construct bacterial microcompartments (BMCs), which are quite rare among studied bacterial lineages. Such BMC genes in planctomycetes are involved in the degradation of plant and algal cell wall sugars and are required for growth on l-fucose, l-rhamnose, and fucoidans (42). Genes involved in l-fucose and l-rhamnose degradation cluster with BMC shell protein-coding genes in the seven *Verrucomicrobia* MAGs (Fig. 4). This is consistent with the high abundance of α-l-fucosidase or α-l-rhamnosidase GH genes (represented by GH29, GH78, GH95, and GH106) in most of these MAGs (Fig. 3), suggesting the importance of fucose- and rhamnose-containing polysaccharides for these *Verrucomicrobia* populations.

**FIG 5:**
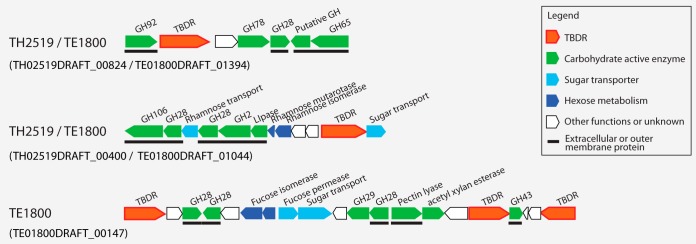
Gene clusters encoding putative *tonB*-dependent carbohydrate utilization (CUT) loci. The IMG gene locus tag for the first gene in each presented gene cluster is indicated in parentheses. The horizontal solid lines below the gene designations indicate predicted extracellular or outer membrane proteins.

TonB-dependent receptor (TBDR) genes were found in *Verrucomicrobia* MAGs and are present at over 20 copies in TE1800 and TH2519. TBDRs are located on the outer cellular membrane of Gram-negative bacteria, usually mediating the transport of iron siderophore complex and vitamin B_12_ across the outer membrane through an active process. More recently, TBDRs were suggested to be involved in carbohydrate transport across the outer membrane by some bacteria that consume complex carbohydrates, and in their carbohydrate utilization (CUT) loci, TBDR genes usually cluster with genes encoding inner membrane transporters, GHs, and regulators for efficient carbohydrate transportation and utilization (43). Such novel CUT loci are present in TE1800 and TH2519, with TBDR genes clustering with genes encoding inner membrane sugar transporters, monosaccharide utilization enzymes, and GHs involved in the degradation of pectin, xylan, and fucose-containing polymers (Fig. 5). Notably, most GHs in the CUT loci are predicted to be extracellular or outer membrane proteins (Fig. 5), catalyzing extracellular hydrolysis reactions to release mono- and oligosaccharides, which are transported across the outer membrane by TBDR proteins. Therefore, such CUT loci may allow these verrucomicrobial populations to coordinately and effectively scavenge the hydrolysis products before they diffuse away.

**FIG 6:**
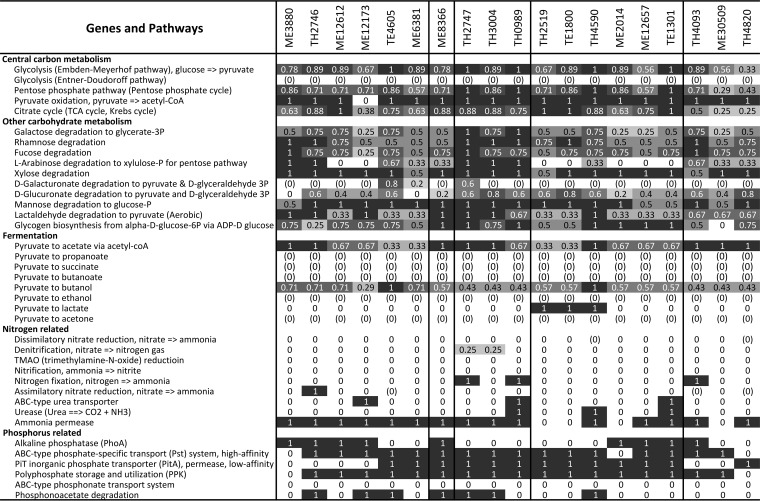
Completeness estimates of key metabolic pathways. Data were shaded in grayscale according to the value, with “1” shaded with the darkest color (black) and “0” with the brightest color (white) to highlight the difference. A completeness value of “1” indicates that a pathway is complete; “0” indicates that no genes were found in that pathway; and “(0)” indicates that, although some genes in a pathway were present, the pathway was likely absent both because signature genes for that pathway were not found in that draft genome and because signature genes were missing in more than two-thirds of all draft genomes.

Genes encoding inner membrane carbohydrate transporters are abundant in *Verrucomicrobia* MAGs (Fig. S4). The Embden-Meyerhof pathway for glucose degradation, as well as pathways for degrading a variety of other sugar monomers, including galactose, rhamnose, fucose, xylose, and mannose, were recovered (in complete or partly complete form) in most MAGs (Fig. 6). As these sugars are abundant carbohydrate monomers in plankton and plant cell walls, the presence of these pathways, together with that of GH genes, suggests that these *Verrucomicrobia* populations may use plankton- and plant-derived saccharides. Machineries for pyruvate degradation to acetyl-coenzyme A (acetyl-CoA) and for the tricarboxylic acid (TCA) cycle are present in most MAGs. These results are largely consistent with their hypothesized role in carbohydrate degradation and previous studies on *Verrucomicrobia* isolates.

10.1128/mSphere.00277-17.5 FIG S4  Counts of carbohydrate and amino acid transporter genes. Download FIG S4, PDF file, 0.1 MB. Copyright © 2017 He et al. 2017 He et al. This content is distributed under the terms of the Creative Commons Attribution 4.0 International license.

Notably, a large number of genes encoding proteins belonging to a sulfatase family (pfam00884) are present in the majority of MAGs (Fig. 2b), which is similar to the high representation of these genes in marine *Verrucomicrobia* genomes (24, 25). Sulfatases hydrolyze sulfate esters, which are rich in sulfated polysaccharides. In general, sulfated polysaccharides are abundant in marine algae and plants (mainly in seaweeds) (44) but have also been found in some freshwater cyanobacteria (45) and plant species (46). Sulfatase genes in our *Verrucomicrobia* MAGs were often located in the same neighborhood as genes encoding extracellular proteins with a putative pectin lyase activity, proteins with a carbohydrate-binding module (pfam13385), GHs, and proteins with planctomycete-specific cytochrome *c* (PSCyt) domains (as shown in Fig. 2c and discussed below). Their genome context lends support to the idea of the participation of these genes in C and sulfur cycling by degrading sulfated polysaccharides, which can serve as an abundant source of sulfur for cell biosynthesis as well as C for energy and growth.

Previously, freshwater *Verrucomicrobia* were suggested to use the algal exudate glycolate in humic lakes, based on the retrieval of genes encoding subunit D (glcD) of glycolate oxidase, which converts glycolate to glyoxylate (26). However, these recovered genes might not be bona fide *glcD* genes due to the lack of other essential subunits as revealed in our study (see Text S1). Among the MAGs, only TE4605 possesses all three essential subunits of glycolate oxidase (*glcDEF*) (Fig. S5). However, genetic context analysis suggests that TE4605 likely uses glycolate for amino acid assimilation instead of for energy generation (Fig. S5 and Text S1). These results are consistent with the absence of the glyoxylate shunt in the 19 *Verrucomicrobia* MAGs, and especially the absence of the malate synthase, which converts glyoxylate to malate to be used through the TCA cycle for energy generation (Fig. S6). Therefore, *Verrucomicrobia* populations represented by the 19 MAGs are not likely key players in glycolate degradation but are more likely important (poly)saccharide degraders in freshwater, as suggested by the high abundances of GH, sulfatase, and carbohydrate transporter genes, metabolic pathways for degrading diverse carbohydrate monomers, and other genome features adapted to the saccharolytic lifestyle.

10.1128/mSphere.00277-17.6 FIG S5  Comparison of glycolate oxidase gene operons in *Escherichia coli*, *Chthoniobacter flavus*, and TE4605. Download FIG S5, PDF file, 0.4 MB. Copyright © 2017 He et al. 2017 He et al. This content is distributed under the terms of the Creative Commons Attribution 4.0 International license.

10.1128/mSphere.00277-17.7 FIG S6  Summary of important metabolic genes and pathways. Download FIG S6, PDF file, 0.1 MB. Copyright © 2017 He et al. 2017 He et al. This content is distributed under the terms of the Creative Commons Attribution 4.0 International license.

### Nitrogen (N) metabolism and adaptation to different N availabilities.

Most *Verrucomicrobia* MAGs in our study did not appear to reduce nitrate or other nitrogenous compounds, and they seemed to take up and use ammonia (Fig. 6), and occasionally amino acids (Fig. S4), as an N source. Further, some Trout Bog populations may have additional avenues to generate ammonia, including genetic machineries for assimilatory nitrate reduction in TH2746, nitrogenase genes for nitrogen fixation, and urease genes in some of the Trout Bog MAGs (Fig. 6), probably as adaptions to N-limited conditions in Trout Bog.

Although Mendota is a eutrophic lake, N can become temporarily limiting during the high-biomass period when N is consumed by large amounts of phytoplankton and bacterioplankton (47). For some bacteria, when N is temporarily limited while C is in excess, cells convert and store the extra C as biopolymers. For example, the verrucomicrobial methanotroph *Methylacidiphilum fumariolicum* SolV accumulated a large amount of glycogen (up to 36% of the total dry weight of cells) when the culture was grown under conditions of N limitation (48). Similarly to this verrucomicrobial methanotroph, genes in glycogen biosynthesis were present in most MAGs from Mendota and Trout Bog (Fig. 6). Indeed, a glycogen synthesis pathway is also present in most genomes of cultivated *Verrucomicrobia* in the public database (data not shown), suggesting that glycogen accumulation might be a common feature for this phylum to cope with the changing pools of C and N in the environment and to facilitate their survival when either is temporally limited.

### Phosphorus (P) metabolism and other metabolic features.

*Verrucomicrobia* populations represented by these MAGs may be able to survive under low-P conditions, as suggested by the presence of genes responding to P limitation, such as the two-component regulator (*phoRB*), alkaline phosphatase (*phoA*), phosphonoacetate hydrolase (*phnA*), and the high-affinity phosphate-specific transporter system (*pstABC*) (Fig. 6). Details of P acquisition and metabolism and other metabolic aspects, such as acetate metabolism, sulfur metabolism, oxygen tolerance, and the presence of the alternative complex III and cytochrome *c* oxidase genes in the oxidative phosphorylation pathway, are discussed in Text S1 and Fig. S6.

### Anaerobic respiration and a putative porin-multiheme cytochrome *c* system.

Respiration using alternative electron acceptors is important for overall lake metabolism in the DOC-rich humic Trout Bog, as the oxygen levels decrease quickly with depth in the water column. We therefore searched for genes involved in anaerobic respiration and found that genes involved in the dissimilatory reduction of nitrate, nitrite, sulfate, sulfite, dimethyl sulfoxide (DMSO), and trimethylamine-N-oxide (TMAO) are largely absent in all MAGs (Fig. S6 and Text S1). Compared to those anaerobic processes, genes for dissimilatory metal reduction are less well understood. In the more extensively studied cultured iron [Fe(III)] reducers, outer surface *c*-type cytochromes (cyt*c*), such as OmcE and OmcS in *Geobacter sulfurreducens*, are involved in Fe(III) reduction at the cell outer surface (49). Further, a periplasmic multiheme cytochrome *c* (MHC; e.g., MtrA in *Shewanella oneidensis* and OmaB/OmaC in *G. sulfurreducens*) can be embedded in a porin (e.g., MtrB in *S. oneidensis* and OmbB/OmbC in *G. sulfurreducens*), forming a porin-MHC complex as an extracellular electron transfer (EET) conduit to reduce extracellular Fe(III) (50, 51). Such outer surface cyt*c* and porin-MHC systems involved in Fe(III) reduction were also suggested to be important in reducing the quinone groups in humic substances (HS) at the cell surface (52–54). The reduced HS can be reoxidized by Fe(III) or oxygen; thus, HS can serve as electron shuttles to facilitate Fe(III) reduction (55, 56) or as regenerable electron acceptors at the anoxic-oxic interface or over redox cycles (57).

**FIG 7:**
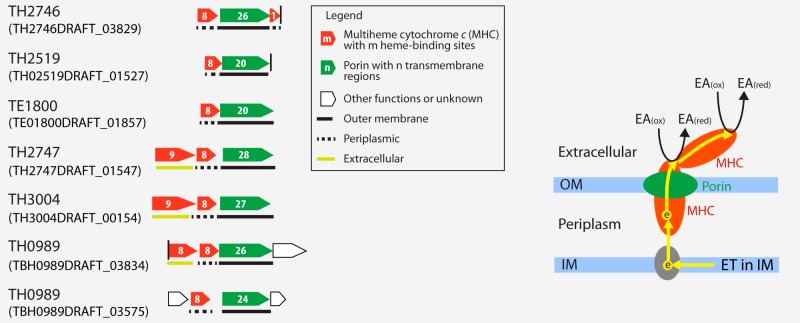
Gene clusters encoding putative porin-multiheme cytochrome *c* complex (PCC). The IMG gene locus tag for the first gene in each presented gene cluster is indicated in parentheses. The vertical line indicates the end of a contig, and horizontal lines below gene designations indicate predicted cellular locations of their encoded proteins. These putative PCC genes are in 18.1-, 9.0-, 6.1-, 18.4-, 70.0-, 10.6-, and 10.8-kbp-long contigs. A hypothesized model of extracellular electron transfer is shown on the right, with yellow arrows indicating electron flows. “IM” and “OM” refer to inner and outer membranes, respectively, “ET in IM” refers to electron transfer in the inner membrane, and “EA_(ox)_” and “EA_(red)_” refer to oxidized and reduced forms of the electron acceptor, respectively.

Outer surface cyt*c* or porin-MHC systems homologous to the ones in *G. sulfurreducens* and *S. oneidensis* are not present in *Verrucomicrobia* MAGs. Instead, we identified a novel porin-coding gene clustering with MHC genes in six MAGs (Fig. 7). These porins were predicted to have at least 20 transmembrane motifs, and their adjacent cyt*c* were predicted to be periplasmic proteins with eight conserved heme-binding sites. In several cases, a gene encoding an extracellular MHC is also located in the same gene cluster. As their gene organization is analogous to that of the porin-MHC gene clusters in *G. sulfurreducens* and *S. oneidensis*, we hypothesize that these genes in *Verrucomicrobia* may encode a novel porin-MHC complex involved in EET.

As these porin-MHC gene clusters are novel, we further confirmed that they are indeed from *Verrucomicrobia*. Their containing contigs were classified to *Verrucomicrobia* based on the consensus of the best BLASTP hits for genes on these contigs. Notably, the porin-MHC gene cluster was observed only in MAGs recovered from the HS-rich Trout Bog, especially from the anoxic hypolimnion environment. Searching the NCBI and IMG databases for the porin-MHC gene clusters homologous to those in Trout Bog, we identified homologs in genomes within the *Verrucomicrobia* phylum, including *Opitutus terrae* PB90-1 isolated from rice paddy soil, *Opitutus* sp. strain GAS368 isolated from forest soil, “*Ca.* Udaeobacter copiosus” recovered from prairie soil, Opititae-40 and Opititae-129 recovered from freshwater sediment, and *Verrucomicrobia* bacterium IMCC26134 recovered from freshwater; some of their residing environments are also rich in HS. Therefore, based on the occurrence pattern of porin-MHC among *Verrucomicrobia* genomes, we hypothesize that such porin-MHCs might participate in EET to HS in anoxic HS-rich environments and that HS may further shuttle electrons to poorly soluble metal oxides or be regenerated at the anoxic-oxic interface, thereby diverting more C flux to respiration instead of fermentation and methanogenesis, which could impact the overall energy metabolism and greenhouse gas emission in the bog environment.

### Occurrence of planctomycete-specific cytochrome *c* and domains.

**FIG 8:**
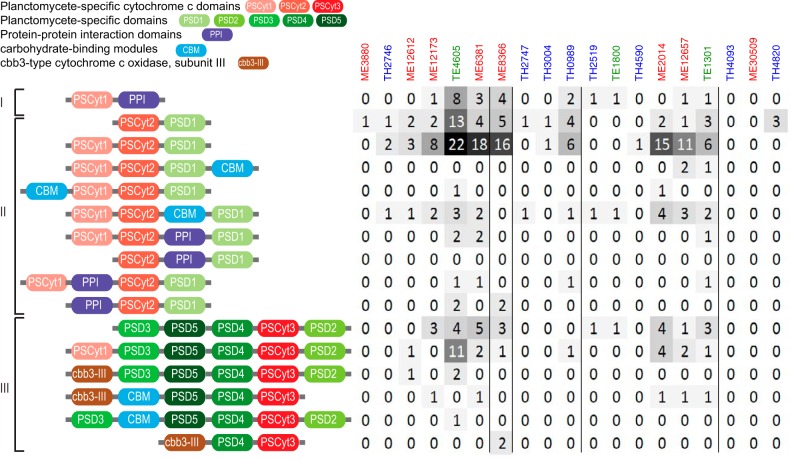
Domain architecture and occurrence of PSCyt-encoding genes. On the basis of combinations of specific PSCyt and PSD domains, these domain structures can be classified into three groups (indicated as I, II, and III). “CBM” refers to carbohydrate-binding modules, which include pfam13385 (Laminin_G_3), pfam08531 (Bac_rhamnosid_N), pfam08305 (NPCBM), pfam03422 (CBM_6), and pfam07691 (PA14). “PPI” refers to protein-protein interaction domains, which include pfam02368 (Big_2), pfam00400 (WD40), and pfam00754 (F5_F8_type_C).

One of the interesting features of *Verrucomicrobia* and its sister phyla in the PVC superphylum is the presence of a number of novel protein domains in some of their member genomes (58, 59). These domains were initially identified in the marine planctomycete *Rhodopirellula baltica* (58) and were therefore referred to as *Planctomycete* specific, although some of them were later identified in other PVC members (59). In our *Verrucomicrobia* MAGs, most genes encoding planctomycete-specific cytochrome *c* domains (PSCyt1 to PSCyt3) also encode other planctomycete-specific domains (PSD1 through PSD5) with various domain combinations and arrangements (Fig. 8 and Fig. S7a). Further, PSCyt2-encoding and PSCyt3-encoding genes are usually next to each of two different families of unknown genes (Fig. S7b). Such conserved domain architectures and gene organizations, as well as their high frequencies of occurrence in some of the *Verrucomicrobia* MAGs, are intriguing, and yet nothing is known about their functions. However, some of the PSCyt-encoding genes also encode protein domains identifiable as carbohydrate-binding modules (CBMs), suggesting a role in carbohydrate metabolism (see detailed discussion in Text S1).

10.1128/mSphere.00277-17.8 FIG S7  Occurrence and gene organization of planctomycete-specific domains, DUF1501, and DUF1552. (a) Counts of PSCyt, PSD, DUF1501, and DUF1552 domains in the MAGs. (b) Clustering of DUF1501- and PSCyt2-encoding genes and clustering of DUF1552- and PSCyt3-encoding genes in the genome. Download FIG S7, PDF file, 0.2 MB. Copyright © 2017 He et al. 2017 He et al. This content is distributed under the terms of the Creative Commons Attribution 4.0 International license.

The coding density of PSCyt-encoding genes indicates that they tend to be more abundant in the epilimnion (either ME or TE) genomes (Fig. 2c), and their coding densities exhibit an inverse correlation with the GH coding density (*r* = −0.62). Interestingly, sulfatase-coding genes are often in the neighborhood of PSCyt-encoding genes in ME and TE genomes, whereas sulfatase-coding genes often neighbor with GH genes in TH genomes. The genomic context suggests that PSCyt-encoding gene functions somewhat mirror those of GH genes (although their reaction mechanisms likely differ fundamentally). However, these PSCyt-encoding gene products were predicted to be periplasmic or cytoplasmic proteins rather than extracellular or outer membrane proteins. Hence, if they are indeed involved in carbohydrate degradation, they likely act on mono- or oligomers that can be transported into the cell. Further, the distribution patterns of GH-encoding genes versus PSCyt-encoding genes between the epilimnion and hypolimnion may reflect the differences in oxygen availability and carbohydrate substrate complexity between the two layers, suggesting some niche differentiation within *Verrucomicrobia* in freshwater systems. Therefore, we suggest that a combination of carbohydrate composition, electron acceptor availability, and C accessibility drives gene distributions in these populations.

### Summary.

The recovery of *Verrucomicrobia* MAGs from the two contrasting lakes greatly expanded the known genomic diversity of freshwater *Verrucomicrobia* and revealed the ecophysiology and some interesting adaptive features of this ubiquitous and yet less-understood freshwater lineage. The overrepresentation of GH, sulfatase, and carbohydrate transporter genes, the genetic potential to use various sugars, and the presence of microcompartments for fucose and rhamnose degradation suggest that they are potentially (poly)saccharide degraders in freshwater. Most of the MAGs encode machineries to cope with the changing availability of N and P and can survive nutrient limitation. Despite these generalities, these *Verrucomicrobia* differ significantly between lakes in the abundance and functional profiles of their GH genes, which may reflect different C sources in the two lakes. Interestingly, a number of MAGs in Trout Bog possess gene clusters potentially encoding a novel porin-multiheme cytochrome *c* complex, which might be involved in extracellular electron transfer in the anoxic humus-rich environment. Intriguingly, large numbers of planctomycete-specific cytochrome *c*-encoding genes are present in MAGs from the epilimnion, exhibiting distribution patterns nearly opposite to those seen with GH genes. Future studies are needed to elucidate the functions of these novel and fascinating genomic features.

In this study, we focused on using genome information to infer the ecophysiology of *Verrucomicrobia*. The rich time-series metagenome data set and the many diverse microbial genomes recovered in these two lakes also provide an opportunity for the future study of *Verrucomicrobia* population dynamics in the context of the total community and their interactions with environmental variables and other microbial groups.

As some of the MAGs analyzed here represent the first genome representatives of several *Verrucomicrobia* subdivisions from freshwater, an interesting issue is whether the populations represented by the MAGs were native aquatic residents and active in aquatic environment or were merely present after having been washed into the lake from surrounding soil. Previous studies on freshwater *Verrucomicrobia* were largely based on analysis of 16S rRNA genes, and yet 16S rRNA genes were not recovered in most MAGs, making it difficult to directly link our MAGs to previously identified freshwater *Verrucomicrobia*. Notably, our MAGs were related only distantly to the ubiquitous and abundant soil *Verrucomicrobia* species “*Ca.* Udaeobacter copiosus” (10) (Fig. 1). In addition, *Verrucomicrobia* were abundant in Trout Bog and other bogs from a 5-year bog lake bacterial community composition and dynamics study (60), with average relative abundances of 7.1% and 8.6% and maximal relative abundances of 25.4% and 39.5% in Trout Bog epilimnion and hypolimnion, respectively. Since the MAGs were presumably from the most abundant *Verrucomicrobia* populations, they were not likely soil immigrants, given their high abundance in the aquatic environment. To confirm their aquatic origin, future experiments should be designed to test their activities and physiology in the aquatic environment on the basis of the genomic insights gained in this study.

## MATERIALS AND METHODS

### Study sites.

Samples for metagenome sequencing were collected from Lake Mendota and Trout Bog Lake, two temperate lakes in Wisconsin in the United States, during the ice-off period (May to November) of each year. Mendota is an urban eutrophic lake with most of its C being autochthonous (produced in-lake), whereas Trout Bog is a small, acidic, and nutrient-poor dystrophic lake with mostly terrestrially derived (allochthonous) C. General lake characteristics are summarized in Table 1.

### Sampling.

For Mendota, we collected depth-integrated water samples from the surface 12 m (mostly consisting of the epilimnion layer) at 94 time points from 2008 to 2012; those samples are referred to as “ME” (38). For Trout Bog, we collected samples from the integrated hypolimnion layer at 45 time points from 2007 to 2009 and samples from the integrated epilimnion layer at 45 time points from 2007 to 2009, and those samples are referred to as “TH” and “TE,” respectively (37). All samples were filtered through 0.22-μm-pore-size polyethersulfone filters and stored at −80°C until extraction. DNA was extracted from the filters using a FastDNA kit (MP Biomedicals) according to the manufacturer’s instructions with some minor modifications as described previously (34).

### Metagenome sequencing and assembly and draft genome recovery.

Details of metagenome sequencing, assembly, and binning were described by Bendall et al. (37) and Hamilton et al. (61). Briefly, shotgun Illumina HiSeq 2500 metagenome libraries were constructed for each of the DNA samples. Three combined assemblies were generated by coassembling reads from all metagenomes within each of the ME, TE, and TH groups. Binning was conducted on the three combined assemblies to recover “metagenome-assembled genomes” (MAGs) based on the combination of contig tetranucleotide frequency and differential coverage patterns across time points using MetaBAT (62). Subsequent manual curation of MAGs was conducted to remove contigs that did not correlate well with the median temporal abundance pattern of all contigs within a MAG, as described by Bendall et al. (37).

### Genome annotation and completeness estimation.

MAGs were submitted to the Department of Energy (DOE) Joint Genome Institute’s Integrated Microbial Genome (IMG) database for gene prediction and function annotation (63). The IMG taxon object identifiers (OIDs) for *Verrucomicrobia* MAGs are listed in Table 2. The completeness and contamination of each MAG were estimated using checkM with both the lineage-specific and *Verrucomicrobia*-specific workflows (35). The *Verrucomicrobia*-specific workflow provided more-accurate estimates (i.e., higher genome completeness and lower contamination) than the lineage-specific workflow in testing performed on 11 complete genomes of *Verrucomicrobia* isolates available at IMG during our method validation. We therefore reported only the estimates from *Verrucomicrobia*-specific workflow (Table 2). MAGs with an estimated completeness level lower than 50% were not included in this study.

### Taxonomic and phylogenetic analysis.

A total of 19 MAGs were classified in the *Verrucomicrobia* phylum based on taxonomic assignment by PhyloSift using 37 conserved phylogenetic marker genes (64), as described by Bendall et al. (37). A phylogenetic tree was reconstructed from the 19 *Verrucomicrobia* MAGs and 25 reference genomes using an alignment concatenated from individual protein alignments of five conserved essential single-copy genes (represented by TIGR01391, TIGR01011, TIGR00663, TIGR00460, and TIGR00362) that were recovered in all *Verrucomicrobia* MAGs. Individual alignments were first generated with MUSCLE (65), concatenated, and trimmed to exclude columns that contained gaps for more than 30% of all sequences. A maximum likelihood phylogenetic tree was constructed using PhyML 3.0 (66), with the LG substitution model and the gamma distribution parameter estimated by PhyML. Bootstrap values were calculated based on 100 replicates. *Kiritimatiella glycovorans* L21-Fru-AB was used as an outgroup in the phylogenetic tree. This bacterium was initially designated the first (and so far the only) cultured representative of *Verrucomicrobia* subdivision 5. However, this subdivision was later proposed as a novel sister phylum associated with *Verrucomicrobia* (67), making it an ideal outgroup for this analysis.

### Estimate of metabolic potential.

IMG provides functional annotation based on KO (KEGG orthology) term, COG (cluster of orthologous group), pfam, and TIGRfam data. To estimate metabolic potential, we primarily used KO terms due to their direct link to KEGG pathways. COG, pfam, and TIGRfam were also used when KO terms were not available for a function. Pathways were primarily reconstructed according to KEGG modules, and the MetaCyc pathway was used if a KEGG module was not available for a pathway. As these MAGs are incomplete genomes, a fraction of genes in a pathway may be missing due to genome incompleteness. Therefore, we estimated the completeness of a pathway as the fraction of recovered enzymes in that pathway (e.g., a pathway is 100% complete if all enzymes in that pathway are encoded by genes recovered in a MAG). As some genes are shared by multiple pathways, signature genes specific for a pathway were used to indicate the presence of a pathway. If signature genes for a pathway were missing in all MAGs, that pathway was likely absent in all genomes. Based on this, we established criteria for estimating pathway completeness in each MAG. If a signature gene in a pathway was present, we report the percentage of genes in the pathway that we found. If a signature gene was absent in a MAG but was present in at least one-third of all MAGs (i.e., ≥7), we still report the pathway completeness for that MAG in order to account for genome incompleteness. Otherwise, we considered the pathway to be absent (i.e., completeness is 0%).

### Glycoside hydrolase identification.

Glycoside hydrolase (GH) genes were identified using the dbCAN annotation tool (http://csbl.bmb.uga.edu/dbCAN/annotate.php) (68) using hmmsearch against hidden Markov models (HMMs) built for all GHs, with an *E* value cutoff of 1e−7, except GH109, for which we found that the HMM used by dbCAN is pfam01408. This pfam is a small domain at the N terminus of GH109 proteins but is not specific for GH109. Therefore, to identify verrucomicrobial GH109, BLASTP was performed using the two GH109 sequences (GenBank accession numbers ACD03864 and ACD04752) from verrucomicrobial *Akkermansia muciniphila* ATCC BAA-835 listed in the CAZy database (http://www.cazy.org), with *E* value cutoff of 1e−6 and a query sequence coverage value cutoff of 50%.

### Other bioinformatic analyses.

Protein cellular location was predicted using CELLO v.2.5 (http://cello.life.nctu.edu.tw) (69) and PSORTb v.3.0 (http://www.psort.org/psortb) (70). The beta-barrel structure of outer membrane proteins was predicted using PRED-TMBB (http://bioinformatics.biol.uoa.gr//PRED-TMBB) (71).
