# Supplementary figures and images for: Ecophysiology of Freshwater Verrucomicrobia Inferred from Metagenome-Assembled Genomes
Source: mSphere. 2017 Sep 27;2(5):e00277-17. doi: 10.1128/mSphere.00277-17 (PMC5615132; doi:10.1128/mSphere.00277-17)

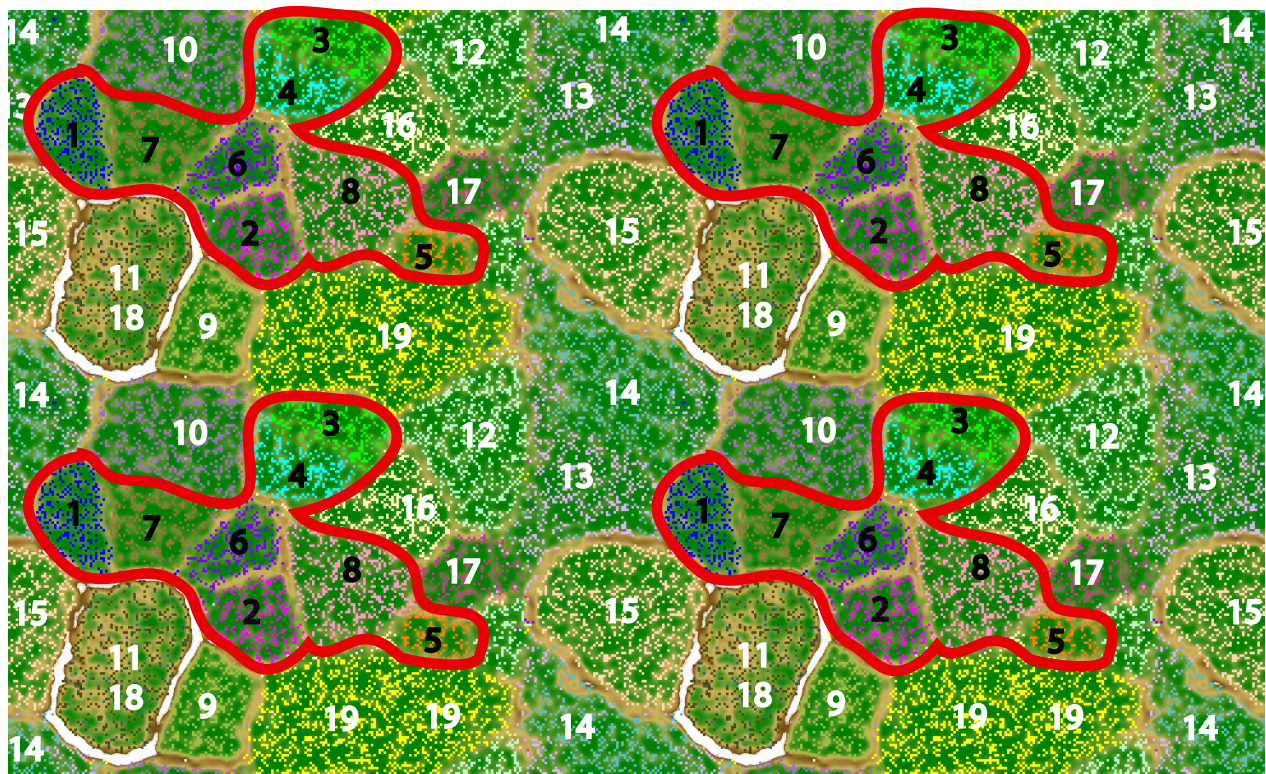

Supplement: FIG S1 [file sph005172368sf2.pdf]

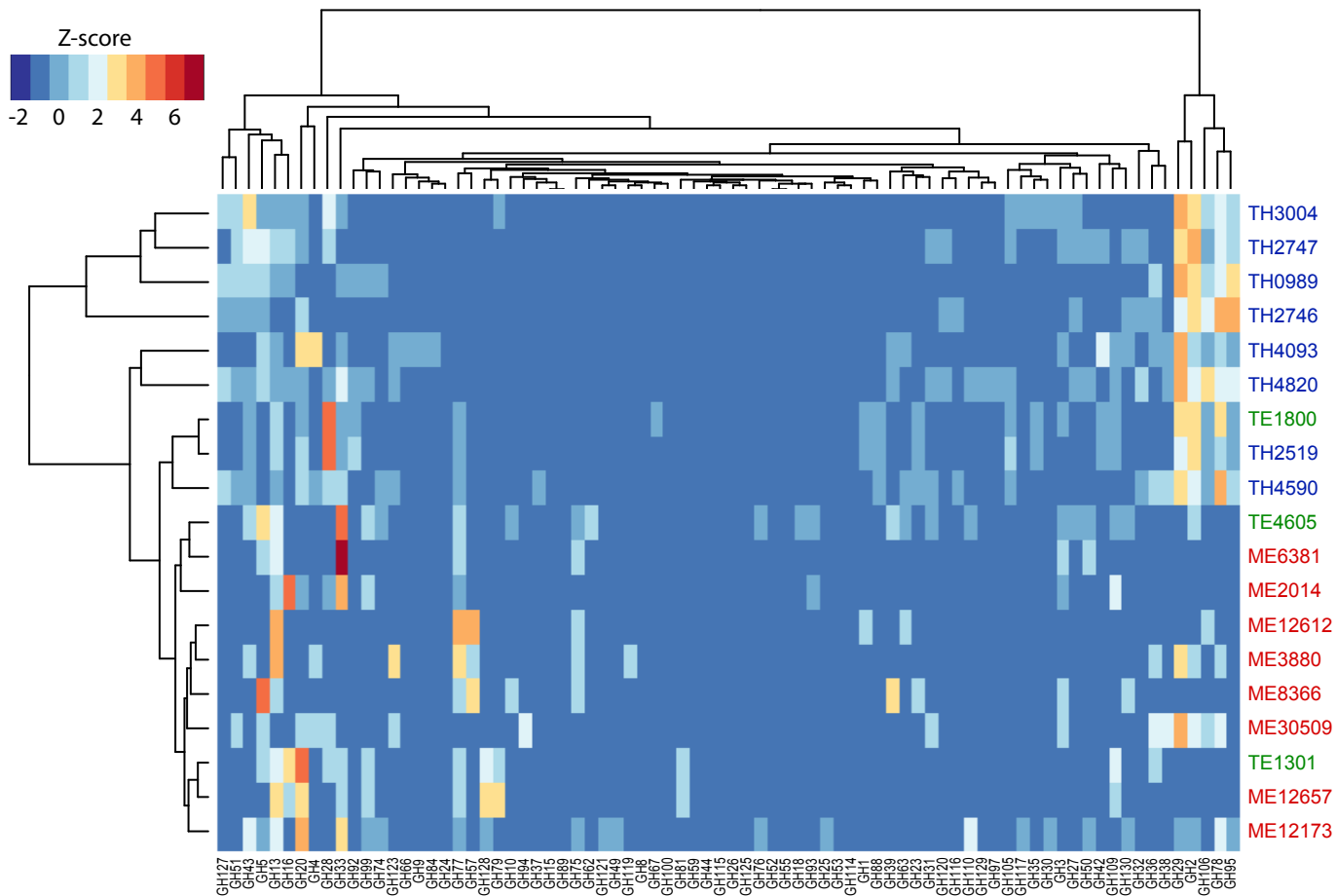

Supplement: FIG S3 [file sph005172368sf4.pdf]
