# Supplementary material for: Ecophysiology of Freshwater Verrucomicrobia Inferred from Metagenome-Assembled Genomes
Source: mSphere. 2017 Sep 27;2(5):e00277-17. doi: 10.1128/mSphere.00277-17 (PMC5615132; doi:10.1128/mSphere.00277-17)
Supplement: TEXT S1 [file sph005172368s1.docx]

**SupplementaL TEXT S1**

**Ecophysiology of Freshwater Verrucomicrobia Inferred from Metagenome-Assembled Genomes**

Shaomei He^1,2^, Sarah L. R. Stevens^1^, Leong-Keat Chan^4^, Stefan Bertilsson^3^, Tijana Glavina del Rio^4^, Susannah G. Tringe^4^, Rex R. Malmstrom^4^, and Katherine D. McMahon^1,5,*^

^1^Department of Bacteriology, University of Wisconsin-Madison, Madison, WI, USA

^2^Department of Geoscience, University of Wisconsin-Madison, Madison, WI, USA

^3^Department of Ecology and Genetics, Limnology and Science for Life Laboratory, Uppsala University, Uppsala, Sweden

^4^DOE Joint Genome Institute, Walnut Creek, CA, USA

^5^Department of Civil and Environmental Engineering, University of Wisconsin-Madison, Madison, WI, USA

* Corresponding author

**Population abundance estimated from MAG coverage depth**

Abundance of populations represented by MAGs at each sampling time point was inferred by the coverage depth of these MAGs within individual metagenomes. First, coverage depth of each contig was obtained by mapping merged reads from each metagenome to all MAGs using the Burrows–Wheeler aligner (BWA)-backtrack alignment algorithm with a 95% sequence identity cutoff and n=0.05, as described in Bendall *et al.* ([1](#_ENREF_1)). Based on the number of reads mapped to each contig, we calculated the coverage depth of each contig. The contig coverage depth was then weighted by its contig length and averaged within each MAG to obtain a weighted average, so that longer contigs (which tend to have more reliable coverage estimation) weigh more in the estimate of the MAG coverage depth. The MAG coverage depth within each metagenome was finally normalized by the total number of reads in each metagenome and multiplied by the maximal number of reads from all metagenomes so that the coverage can be compared across different time points and different lakes (**Table 2**).

**Glycolate utilization**

Previously, Verrucomicrobia were suggested to be among the glycolate utilizers in humic lakes, based on the retrieval of genes encoding glycolate oxidase subunit D (*glcD*) ([2](#_ENREF_2)). Glycolate is an algal exudate, which was suggested to influence bacterial community structure in lakes. The first step in bacterial glycolate utilization is converting glycolate to glyoxylate by glycolate oxidase, a multi-subunit protein complex consisted of subunits D, E, and F (*glcDEF*). In *E. coli*, all three subunits are essential to its activity ([3](#_ENREF_3)). The *glc* operon of *E. coli* also contains *glcB*, encoding malate synthase G, which converts glyoxylate to malate to be utilized through the TCA cycle. Among the MAGs, only TE4605 possesses all three subunits of glycolate oxidase (*glcDEF)* (**Fig. S5**). However, different from *E. coli*, the TE4605 *glc* operon lacks the malate synthase G, but contains an alanine (or serine)-glyoxylate transaminase (AGXT) and a glycolate permease instead (**Fig. S5**). Therefore, it is likely that glyoxylate generated from glycolate oxidation is converted to glycine for amino acid assimilation (**Fig. S5**), instead of energy generation through the TCA cycle as in *E. coli*. A similar operon containing *glcDEF* and AGXT is also present in a soil verrucomicrobial aerobe, *Chthoniobacter flavus*. Notably, *C. flavus* was reported unable to grow on glycolate as the sole carbon and energy source ([4](#_ENREF_4)), supporting our hypothesis that glycolate is likely utilized for amino acid assimilation, instead of energy generation by TE4605.

Interestingly, TE4605 also contains a second copy of *glcD* (*glcD*2), which is not associated with *glcEF*. *GlcD*2 only shares a 34% amino acid identity with *glcD* in the *glc* operon mentioned earlier. Notably, this *glcD*2 is 100% identical at the nucleotide level to the verrucomicrobial *glcD* clone OTU45 from the study by Paver and Kent ([2](#_ENREF_2)) likely derived from the same species. In fact, nearly all MAGs have *glcD*, some of which share >60% amino acid identities to *glcD* clones (OTU43, OTU44 and OTU45). However, these *glcD*, like *glcD*2 in TE4605, lack *glcEF* in its genome vicinity, and *glcD* is either an orphan gene or on operons that are not apparently involved in glycolate metabolism. Therefore, these genes are not likely bona fide *glcD*. Overall, the lack of glycolate oxidase is consistent with the absence of glyoxylate cycle and especially the malate synthase, which converts glyoxylate to malate to be utilized through the TCA cycle for energy generation in the 19 Verrucomicrobia MAGs (**Fig. S6**). Therefore, Verrucomicrobia populations represented by the 19 MAGs are not likely key players in glycolate degradation

**Acetate metabolism**

Transporters for monocarboxylic acid (such as pyruvate, acetate, propionate) belong to a large solute:sodium symporter (SSS) family, which can transport sugars, amino acids, nucleosides, inositols, vitamins, urea or anions. Genes belong to SSS family were present in all MAGs, yet most of their substrate specificities based on the annotation are unknown. Several SSS genes are annotated as acetate permeases (actP), together with the presence of genes for acetate activation to acetyl-CoA in these MAGs, actP would allow acetate enter the TCA cycle for energy generation. However, these MAGs lack isocitrate lyase and malate synthase (**Fig. S6**), key enzymes on the glyoxylate cycle, which is necessary when cells grow with two-carbon compounds, such as acetate as the sole carbon source. Pathways alternative to the glyoxylate shunt have been proposed to replenish four-carbon intermediates during growth on acetate. Yet, among the MAGs, only TH2746 possess key genes in the ethylmalonyl-CoA pathway for growing on acetate (**Fig. S6**) ([5](#_ENREF_5)). Therefore, for most MAG-represented freshwater Verrucomicrobial populations, acetate might be used as a supplementary source of energy, but not as the sole energy and carbon source for growth.

**Phosphorus (P) metabolism and adaptation to P-limited conditions**

The high-affinity phosphate-specific transporter (PstABC) system genes were recovered in nearly all MAGs, and the low-affinity phosphate permease (PitA) genes are also present in most MAGs (**Fig. 7**), allowing cells to efficiently take up inorganic phosphate at a wide range of concentrations. In addition, alkaline phosphatase (PhoA) genes were recovered in half of the MAGs and phosphonoacetate hydrolase (PhnA) genes are also present in some MAGs. These two enzymes enable cells to use phosphate monoesters and organophosphonates as a P source under P starvation, respectively. Further, the polyphosphate kinase (PPK) genes found in nearly all MAGs may allow cells to accumulate polyphosphate for future use when environmental P becomes scarce. Overall, the presence of genes responding to P limitation, such as the two-component regulator (*phoRB*), *phoA*, *phnA*, and *pstABC* in these Verrucomicrobia populations suggest a strategy to survive P limitation. Previously, positive correlations between freshwater Verrucomicrobia abundance and P availability were observed ([6](#_ENREF_6), [7](#_ENREF_7)). However, despite the much higher P levels in Mendota, we did not observe higher population abundance of Verrucomicrobia in Mendota or underrepresentation of their genes responding to P limitation. Therefore, Verrucomicrobia populations in Mendota are probably more influenced by the availability of organic autochthonous C substrates.

**Sulfur metabolisms**

Dissimilatory sulfate reduction genes were only found in TH4590, and genes for Dimethylsulfoxide (DMSO) reduction and polysulfide reduction are absent in all MAGs, as are sulfur and thiosulfate oxidation (SOX) genes (**Fig. S6**). These suggest that redox processes with sulfur-containing compounds are not important modes of energy generation for these Verrucomicrobia populations.

The ABC-type sulfate transporter or sulfate permease genes, as well as assimilatory sulfate reduction genes were found in most MAGs (**Fig. S6**). By contrast, sulfonate transporter genes were only found in TH4903, and genes encoding alkanesulfonate monooxygenase, which is involved in sulfur acquisition under sulfur-limiting conditions by splitting organosulfonates to sulfite and formaldehyde, are absent in all MAGs. The presence of sulfate transporter and assimilatory sulfate reduction genes, and the absence of genes involved in sulfur acquisition under sulfur-limited conditions is consistent with our hypothesis that the degradation of sulfated polysaccharide may serve as an abundant source of sulfur for cell biosynthesis, based on the high occurrence of sulfatase genes in these MAGs.

**Oxygen tolerance**

Oxygen (O­_2_) reduction products such as superoxide (O_2_^-^) and hydrogen peroxide (H_2_O_2_) can damage cells. Superoxide dismutases (SODs) convert O_2_^-^ to H_2_O_2_ and O­_2_, and H_2_O_2_ is less destructive to cells and can be subsequently eliminated by the activities of catalases or peroxidases. All MAGs have SOD genes, and the majority of them also contain catalase and/or peroxidase genes (**Fig. S6**). The lack of catalases and peroxidase is not particularly associated with MAGs recovered from the anoxic hypolimnion, but rather is probably due to the incomplete coverage of their genomes. Therefore, the presence of SOD, catalase and/or peroxidase genes in most of MAGs suggests that most of these Verrucomicrobia, including the ones found in hypolimnion, are able to tolerate oxygen.

**Oxidative phosphorylation and alternative complex III**

Most of these MAGs possess genetic components of the oxidative phosphorylation pathway, including NADH:quinone oxidoreductase (Complex I), succinate:quinone oxidoreductase (Complex II), the low-affinity *caa_3_*-type cytochrome *c* oxidase and/or the high-affinity *cbb_3_*-type cytochrome *c* oxidase (Complex IV), and the F-ATPase (Complex V) (**Fig. S6**). However, bona fide cytochrome *bc_1_* complex, an quinol:cytochrome *c* oxidoreductase (Complex III), is missing in all of the MAGs, and is also missing in all Verrucomicrobia isolate genomes, including the obligate aerobes (data not shown). An alternative complex III (ACIII) was proposed to perform the same function traditionally provided by cytochrome *bc_1_* complex in Bacteroidetes *Rhodothermus marinus* ([8](#_ENREF_8)). We found ACIII genes in Verrucomicrobia isolate genomes and most MAGs, suggesting this phylum uses ACIII for electron transfer. In some cases, ACIII is immediately upstream of *cbb_3_*-type cytochrome *c* oxidase complex located in the same operon in some cases. Taken together, the presence of oxidative phosphorylation and cytochrome *c* oxidase genes would enable oxygen to be used as an electron acceptor for energy generation. Interestingly, the low-affinity *aa_3_*-type cytochrome c oxidase genes are not restricted to MAGs in the epilimnion where oxygen is available in higher concentrations.

**Occurrence of Planctomycete-specific cytochrome *c* and domains**

A number of domains that were initially identified as “Planctomycete-specific” ([9](#_ENREF_9)) are abundant in our Verrucomicrobia MAGs. Among them are three Planctomycete-specific cytochrome *c* domains (PSCyt1, PSCyt2, and PSCyt3, represented by pfam07635, pfam07583, and pfam07627, respectively), five Planctomycete-specific domains (PSD1 through PSD5, represented by pfam07587, pfam07624, pfam07626, pfam07631, and pfam07637, respectively), and two domains with unknown functions (DUF1501 and DUF1552, represented by pfam07394 and pfam07586, respectively). PSCyt-encoding genes in our Verrucomicrobia MAGs encode multi-domain proteins, most of which contain both PSCyt and PSD domains and exhibit various domain architectures. Based on the combination of specific PSCyt and PSD, these domain structures can be classified into three groups. Group I contains PSCyt1, but not PSD or other PSCyt; Group II contains PSCyt2, which exclusively pairs with PSD1 and also often with PSCyt1; and Group III contains PSCyt3, which exclusively pairs with PSD4 and also often with PSD2, PSD3 and PSD5 (**Fig. 8**). The pairing between specific PSCyt and PSD is also reflected in their domain occurrence frequencies in these MAGs (**Fig. S7a**). Further, PSCyt2-encoding genes are usually next to DUF1501-encoding genes; and PSCyt3-encoding genes are usually next to DUF1552-encoding genes (**Fig. S7b**). Such conserved domain architectures and gene organizations, as well as their high occurrence frequencies in some of the Verrucomicrobia MAGs are intriguing, yet nothing is known about their functions.

Some of the PSCyt-encoding genes also encode additional domains besides PSCyt and PSD. Most of these additional domains can be classified into two categories: one involved in protein-protein interactions (PPI) and the other involved in carbohydrate binding (CBM, carbohydrate-binding modules) (**Fig. 8**), similar to previous findings in a number of PVC genomes by Kamneva *et al.* ([10](#_ENREF_10)). These authors suggested that PPI domains in these genes were responsible for protein complex assembly or substrate recognition, and cytochromes encoded by these genes likely transfer electrons to acceptors (possibly proteins and sugars) due to the presence of CBM domains ([10](#_ENREF_10)). The presence of CBM domains in redox active proteins is indeed interesting. For example, both CBM1 and cytochrome *b*562 (another redox active protein domain) are components of cellobiose dehydrogenase (CDH) in the white-rot fungus *Phanerochaete chrysosporium* ([11](#_ENREF_11)) and sugar dehydrogenase (SDH) in mushroom *Coprinopsis cinerea* ([12](#_ENREF_12)). Therefore, it is plausible that some of the PSCyt-encoding genes, especially the ones with CBMs, are involved in carbohydrate degradation.

**REFERENCES**

1. **Bendall ML, Stevens SLR, Chan L-K, Malfatti S, Schwientek P, Tremblay J, Schackwitz W, Martin J, Pati A, Bushnell B, Froula J, Kang D, Tringe SG, Bertilsson S, Moran MA, Shade A, Newton RJ, McMahon KD, Malmstrom RR**. 2016. Genome-wide selective sweeps and gene-specific sweeps in natural bacterial populations. ISME J doi:10.1038/ismej.2015.241.

2. **Paver SF, Kent AD**. 2010. Temporal patterns in glycolate-utilizing bacterial community composition correlate with phytoplankton population dynamics in humic lakes. Microb Ecol **60**:406-18.

3. **Pellicer MT, Badia J, Aguilar J, Baldoma L**. 1996. glc locus of Escherichia coli: characterization of genes encoding the subunits of glycolate oxidase and the glc regulator protein. J Bacteriol **178**:2051-9.

4. **Sangwan P, Chen X, Hugenholtz P, Janssen PH**. 2004. Chthoniobacter flavus gen. nov., sp. nov., the first pure-culture representative of subdivision two, Spartobacteria classis nov., of the phylum Verrucomicrobia. Appl Environ Microbiol **70**:5875-81.

5. **Schneider K, Peyraud R, Kiefer P, Christen P, Delmotte N, Massou S, Portais JC, Vorholt JA**. 2012. The ethylmalonyl-CoA pathway is used in place of the glyoxylate cycle by Methylobacterium extorquens AM1 during growth on acetate. J Biol Chem **287**:757-66.

6. **Lindström ES, Vrede K, Leskinen E**. 2004. Response of a member of the Verrucomicrobia, among the dominating bacteria in a hypolimnion, to increased phosphorus availability. Journal of Plankton Research **26**:241-246.

7. **Haukka K, Kolmonen E, Hyder R, Hietala J, Vakkilainen K, Kairesalo T, Haario H, Sivonen K**. 2006. Effect of nutrient loading on bacterioplankton community composition in lake mesocosms. Microb Ecol **51**:137-46.

8. **Pereira MM, Refojo PN, Hreggvidsson GO, Hjorleifsdottir S, Teixeira M**. 2007. The alternative complex III from Rhodothermus marinus - a prototype of a new family of quinol:electron acceptor oxidoreductases. FEBS Lett **581**:4831-5.

9. **Studholme DJ, Fuerst JA, Bateman A**. 2004. Novel protein domains and motifs in the marine planctomycete Rhodopirellula baltica. FEMS Microbiol Lett **236**:333-40.

10. **Kamneva OK, Knight SJ, Liberles DA, Ward NL**. 2012. Analysis of genome content evolution in pvc bacterial super-phylum: assessment of candidate genes associated with cellular organization and lifestyle. Genome Biol Evol **4**:1375-90.

11. **Yoshida M, Igarashi K, Wada M, Kaneko S, Suzuki N, Matsumura H, Nakamura N, Ohno H, Samejima M**. 2005. Characterization of carbohydrate-binding cytochrome b562 from the white-rot fungus Phanerochaete chrysosporium. Appl Environ Microbiol **71**:4548-55.

12. **Matsumura H, Umezawa K, Takeda K, Sugimoto N, Ishida T, Samejima M, Ohno H, Yoshida M, Igarashi K, Nakamura N**. 2014. Discovery of a Eukaryotic Pyrroloquinoline Quinone-Dependent Oxidoreductase Belonging to a New Auxiliary Activity Family in the Database of Carbohydrate-Active Enzymes. PLoS ONE **9**:e104851.
