# Supplementary material for: Ecophysiology of Freshwater Verrucomicrobia Inferred from Metagenome-Assembled Genomes
Source: mSphere. 2017 Sep 27;2(5):e00277-17. doi: 10.1128/mSphere.00277-17 (PMC5615132; doi:10.1128/mSphere.00277-17)
Supplement: FIG S2 [file sph005172368sf3.pdf]

GH families	ME3880	TH2746	ME12612	ME12173	TE4605	ME6381	ME8366	TH2747	TH3004	TH0989	TH2519	TE1800	TH4590	ME2014	ME12657	TE1301	TH4093	ME30509	TH4820
GH29	2	22	0	1	0	0	0	17	19	24	4	5	6	0	0	0	14	3	13
GH2	1	24	0	1	2	0	0	18	14	20	5	5	5	0	0	0	6	2	7
GH78	1	31	0	2	0	0	0	11	11	13	3	5	9	0	0	0	5	2	7
GH95	0	29	0	1	0	0	0	7	9	17	1	2	3	0	0	0	4	0	7
GH106	0	21	1	0	0	0	0	4	9	12	1	2	1	0	0	0	3	1	10
GH13	3	3	2	2	3	2	1	7	6	7	2	3	2	2	2	2	3	0	3
GH20	0	14	0	4	0	0	0	3	5	2	2	2	3	1	2	4	10	1	3
GH5	0	4	0	1	4	1	3	13	4	10	0	0	0	0	0	1	6	1	6
GH28	0	1	0	0	0	0	0	7	11	2	8	8	3	1	0	1	0	1	3
GH43	1	4	0	2	2	0	0	11	15	9	1	1	2	0	0	0	0	0	2
GH33	0	1	0	3	7	6	0	1	3	5	1	1	4	5	1	1	3	0	8
GH127	0	9	0	0	0	0	0	2	7	8	0	0	4	0	0	0	0	0	6
GH16	0	1	0	0	0	0	0	8	6	7	0	0	0	6	1	3	0	0	3
GH51	0	5	0	0	0	0	0	7	7	9	0	0	1	0	0	0	1	1	2
GH36	1	4	0	0	0	0	0	1	1	8	0	0	4	0	0	1	3	2	1
GH3	0	2	0	0	1	1	1	6	4	3	0	1	0	1	0	0	2	1	0
GH32	0	6	0	0	0	0	0	6	0	3	0	0	2	0	0	0	0	0	6
GH77	2	2	2	1	2	1	1	1	1	1	1	1	1	1	1	1	0	0	0
GH109	0	3	0	0	1	0	0	1	1	1	1	1	0	3	1	2	4	0	2
GH42	0	1	0	0	0	0	0	4	1	3	1	2	0	0	0	0	7	0	1
GH27	0	4	0	0	1	0	0	5	3	3	0	0	0	0	0	0	0	0	2
GH50	0	1	0	1	1	1	0	5	2	3	0	0	0	0	0	0	0	0	4
GH99	0	0	0	1	2	0	0	1	0	5	0	0	0	2	1	1	1	0	4
GH105	0	1	0	0	0	0	0	4	5	0	2	2	1	0	0	0	0	0	2
GH130	0	4	0	1	1	0	1	4	1	1	0	0	0	0	0	0	4	0	1
GH38	0	2	0	0	0	0	0	0	1	3	0	0	4	0	0	0	4	2	3
GH39	0	1	0	0	2	0	2	2	2	2	0	0	0	0	0	0	2	0	3
GH4	1	1	0	0	0	0	0	0	0	0	0	0	2	0	0	0	12	1	0
GH74	0	1	0	1	1	0	0	2	1	6	0	0	1	0	0	0	1	0	1
GH92	0	1	0	0	0	0	0	2	0	4	2	2	0	0	0	0	0	0	3
GH120	0	4	0	0	0	0	0	3	1	2	0	0	0	0	0	0	0	0	2
GH35	0	0	0	0	0	0	0	2	5	2	1	1	0	0	0	0	1	0	1
GH23	0	0	0	0	0	0	1	2	1	2	1	1	2	0	0	0	1	0	1
GH110	0	2	0	2	1	0	0	1	0	2	0	0	0	0	0	0	0	0	3
GH116	0	5	0	0	0	0	0	1	1	2	0	0	1	0	0	0	1	0	1
GH31	0	1	0	0	1	0	0	3	1	1	0	0	1	0	0	0	1	1	2
GH63	0	1	1	0	1	0	0	2	0	2	0	0	1	0	0	0	3	0	0
GH30	0	1	0	1	0	0	0	2	5	2	0	0	0	0	0	0	0	0	0
GH57	1	1	2	0	0	0	2	1	1	2	0	0	0	0	0	0	0	0	1
GH123	2	0	0	0	0	0	0	0	0	1	0	0	1	0	0	0	3	1	2
GH128	0	0	0	1	0	0	0	1	2	0	0	0	0	0	2	2	0	0	1
GH129	0	3	0	0	0	0	0	0	0	2	0	0	0	0	0	0	1	0	2
GH1	0	0	1	0	0	0	0	1	1	0	1	1	0	0	0	0	1	0	1
GH10	0	3	0	0	1	0	1	1	1	1	0	0	0	0	0	0	0	0	0
GH117	0	1	0	1	0	0	0	1	3	1	0	0	0	0	0	0	0	0	1
GH75	1	1	1	1	1	1	1	0	0	0	0	0	0	0	0	0	0	0	0
GH79	0	0	0	0	0	0	0	0	3	1	0	0	0	0	2	1	0	0	0
GH88	0	0	0	0	0	0	0	0	1	1	1	1	1	0	0	0	0	0	1
GH94	0	2	0	0	0	0	0	0	0	1	0	0	0	0	0	0	1	2	0
GH97	0	2	0	0	0	0	0	0	0	3	0	0	0	0	0	0	0	0	2
GH76	0	1	0	1	1	0	0	1	0	1	0	0	0	0	0	0	0	0	1
GH44	0	0	0	0	0	0	0	1	1	2	0	0	0	0	0	0	0	0	1
GH9	0	1	0	0	0	0	0	1	0	1	0	0	0	0	0	0	2	0	0
GH115	0	0	0	0	0	0	0	1	1	1	0	0	0	0	0	0	0	0	1
GH25	0	0	0	1	0	0	0	1	2	0	0	0	0	0	0	0	0	0	0
GH37	0	2	0	0	0	0	0	0	0	0	0	0	1	0	0	0	1	0	0
GH59	0	1	0	0	0	0	0	0	1	2	0	0	0	0	0	0	0	0	0
GH66	0	0	0	0	0	0	0	0	0	0	0	0	0	0	0	0	4	0	0
GH81	0	0	0	0	0	0	0	1	0	1	0	0	0	0	1	1	0	0	0
GH15	0	2	0	0	0	0	0	0	1	0	0	0	0	0	0	0	0	0	0
GH18	0	0	0	0	1	0	0	0	0	1	0	0	0	0	0	0	1	0	0
GH26	0	0	0	0	0	0	0	1	0	2	0	0	0	0	0	0	0	0	0
GH53	0	0	0	0	0	0	0	1	1	1	0	0	0	0	0	0	0	0	0
GH84	0	0	0	0	0	0	0	0	1	0	0	0	0	0	0	0	2	0	0
GH89	0	2	0	0	0	0	0	0	1	0	0	0	0	0	0	0	0	0	0
GH93	0	0	0	0	1	0	0	0	0	1	0	0	0	1	0	0	0	0	0
GH114	0	0	0	0	0	0	0	1	1	0	0	0	0	0	0	0	0	0	0
GH119	1	0	0	0	0	0	0	1	0	0	0	0	0	0	0	0	0	0	0
GH121	0	0	0	1	0	0	0	0	1	0	0	0	0	0	0	0	0	0	0
GH125	0	0	0	0	0	0	0	1	0	1	0	0	0	0	0	0	0	0	0
GH62	0	0	0	0	2	0	0	0	0	0	0	0	0	0	0	0	0	0	0
GH67	0	0	0	0	0	0	0	0	0	0	0	1	0	0	0	0	0	0	0
GH100	0	0	0	0	0	0	0	0	0	0	0	0	0	0	0	0	0	0	1
GH24	0	0	0	0	0	0	0	0	0	0	0	0	0	0	0	0	1	0	0
GH49	0	0	0	1	0	0	0	0	0	0	0	0	0	0	0	0	0	0	0
GH52	0	0	0	0	0	0	0	0	0	1	0	0	0	0	0	0	0	0	0
GH55	0	0	0	0	0	0	0	0	0	1	0	0	0	0	0	0	0	0	0
GH8	0	0	0	0	0	0	0	1	0	0	0	0	0	0	0	0	0	0	0
