# Supplementary material for: Ecophysiology of Freshwater Verrucomicrobia Inferred from Metagenome-Assembled Genomes
Source: mSphere. 2017 Sep 27;2(5):e00277-17. doi: 10.1128/mSphere.00277-17 (PMC5615132; doi:10.1128/mSphere.00277-17)
Supplement: FIG S4 [file sph005172368sf5.pdf]

| COG ID | Function name | ME3880 | TH2746 | ME12612 | ME12173 | TE4605 | ME6381 | ME8366 | TH2747 | TH3004 | TH0989 | TH2519 | TE1800 | TH4590 | ME2014 | ME12657 | TE1301 | TH4093 | ME30509 | TH4820 |
|---|---|---|---|---|---|---|---|---|---|---|---|---|---|---|---|---|---|---|---|---|
| **Carbohydrate transport and metabolism** | | | | | | | | | | | | | | | | | | | | |
| COG2814 | Arabinose efflux permease | 0 | 11 | 2 | 2 | 9 | 1 | 7 | 10 | 5 | 10 | 4 | 6 | 6 | 2 | 4 | 4 | 3 | 0 | 6 |
| COG2271 | Sugar phosphate permease | 0 | 1 | 0 | 2 | 3 | 5 | 5 | 5 | 4 | 8 | 6 | 5 | 4 | 4 | 1 | 3 | 0 | 0 | 2 |
| COG0738 | Fucose permease | 2 | 2 | 0 | 0 | 1 | 2 | 0 | 4 | 2 | 3 | 3 | 3 | 1 | 2 | 1 | 2 | 0 | 0 | 3 |
| COG1762 | Phosphotransferase system mannitol/fructose-specific IIA domain (Ntr-type) | 2 | 1 | 1 | 1 | 1 | 0 | 1 | 2 | 1 | 4 | 1 | 1 | 3 | 0 | 0 | 1 | 4 | 3 | 2 |
| COG2211 | Na+/melibiose symporter and related transporters | 1 | 2 | 0 | 0 | 0 | 0 | 1 | 5 | 5 | 3 | 0 | 1 | 0 | 0 | 0 | 0 | 7 | 0 | 0 |
| COG1172 | Ribose/xylose/arabinose/galactoside ABC-type transport systems, permease components | 0 | 1 | 0 | 2 | 1 | 1 | 4 | 2 | 2 | 3 | 0 | 0 | 0 | 1 | 1 | 0 | 0 | 2 | 0 |
| COG1879 | ABC-type sugar transport system, periplasmic component | 0 | 0 | 0 | 1 | 0 | 2 | 3 | 3 | 2 | 2 | 0 | 0 | 2 | 1 | 1 | 1 | 0 | 1 | 0 |
| COG0580 | Glycerol uptake facilitator and related permeases | 1 | 1 | 2 | 0 | 2 | 1 | 1 | 1 | 0 | 2 | 1 | 1 | 0 | 2 | 1 | 2 | 1 | 0 | 0 |
| COG1129 | ABC-type sugar transport system, ATPase component | 0 | 1 | 0 | 1 | 1 | 1 | 3 | 1 | 1 | 2 | 1 | 1 | 0 | 1 | 1 | 0 | 0 | 1 | 0 |
| COG1925 | Phosphotransferase system, HPr-related proteins | 1 | 1 | 1 | 0 | 1 | 0 | 1 | 1 | 1 | 0 | 1 | 1 | 1 | 1 | 1 | 1 | 1 | 1 | 1 |
| COG1653 | ABC-type sugar transport system, periplasmic component | 0 | 1 | 0 | 0 | 0 | 0 | 0 | 0 | 1 | 0 | 0 | 0 | 10 | 0 | 0 | 0 | 1 | 0 | 1 |
| COG1682 | ABC-type polysaccharide/polyol phosphate export systems, permease component | 1 | 0 | 0 | 0 | 0 | 0 | 0 | 0 | 1 | 1 | 0 | 0 | 2 | 0 | 0 | 0 | 0 | 0 | 2 |
| COG1134 | ABC-type polysaccharide/polyol phosphate transport system, ATPase component | 1 | 0 | 0 | 0 | 0 | 0 | 0 | 0 | 1 | 1 | 0 | 0 | 2 | 0 | 0 | 0 | 0 | 0 | 2 |
| COG0395 | ABC-type sugar transport system, permease component | 0 | 0 | 0 | 0 | 0 | 0 | 0 | 0 | 0 | 0 | 0 | 0 | 4 | 0 | 0 | 0 | 1 | 0 | 0 |
| COG3839 | ABC-type sugar transport systems, ATPase components | 0 | 0 | 0 | 0 | 0 | 0 | 0 | 0 | 0 | 1 | 1 | 1 | 1 | 0 | 0 | 0 | 1 | 0 | 0 |
| COG4213 | ABC-type xylose transport system, periplasmic component | 0 | 0 | 0 | 0 | 1 | 1 | 0 | 0 | 0 | 0 | 1 | 1 | 1 | 0 | 0 | 0 | 0 | 0 | 0 |
| COG1869 | ABC-type ribose transport system, auxiliary component | 0 | 1 | 0 | 0 | 0 | 0 | 0 | 1 | 1 | 1 | 0 | 0 | 0 | 0 | 0 | 0 | 0 | 0 | 0 |
| COG1175 | ABC-type sugar transport systems, permease components | 0 | 0 | 0 | 0 | 0 | 0 | 0 | 0 | 0 | 0 | 0 | 0 | 3 | 0 | 0 | 0 | 1 | 0 | 0 |
| COG4214 | ABC-type xylose transport system, permease component | 0 | 0 | 0 | 0 | 1 | 0 | 0 | 0 | 0 | 0 | 1 | 1 | 1 | 0 | 0 | 0 | 0 | 0 | 0 |
| COG2610 | H+/gluconate symporter and related permeases | 0 | 0 | 0 | 0 | 0 | 1 | 0 | 0 | 0 | 0 | 0 | 1 | 0 | 0 | 1 | 1 | 0 | 0 | 0 |
| COG1593 | TRAP-type C4-dicarboxylate transport system, large permease component | 0 | 0 | 0 | 0 | 0 | 0 | 0 | 0 | 0 | 1 | 0 | 0 | 1 | 0 | 0 | 0 | 0 | 0 | 0 |
| COG1638 | TRAP-type C4-dicarboxylate transport system, periplasmic component | 0 | 0 | 0 | 0 | 0 | 0 | 0 | 0 | 0 | 1 | 0 | 0 | 1 | 0 | 0 | 0 | 0 | 0 | 0 |
| COG1445 | Phosphotransferase system fructose-specific component IIB | 0 | 0 | 0 | 0 | 0 | 0 | 0 | 0 | 0 | 1 | 0 | 0 | 0 | 0 | 0 | 0 | 0 | 0 | 0 |
| COG1299 | Phosphotransferase system, fructose-specific IIC component | 0 | 0 | 0 | 0 | 0 | 0 | 0 | 0 | 0 | 1 | 0 | 0 | 0 | 0 | 0 | 0 | 0 | 0 | 0 |
| COG4975 | Putative glucose uptake permease | 0 | 0 | 0 | 0 | 0 | 0 | 0 | 0 | 0 | 1 | 0 | 0 | 0 | 0 | 0 | 0 | 0 | 0 | 0 |
| **Sum** | | **9** | **23** | **6** | **9** | **21** | **15** | **26** | **35** | **27** | **46** | **20** | **23** | **43** | **14** | **12** | **15** | **20** | **8** | **19** |
| **Amino acid transport and metabolism** | | | | | | | | | | | | | | | | | | | | |
| COG0591 | Na+/proline symporter | 2 | 0 | 2 | 1 | 1 | 2 | 2 | 2 | 4 | 5 | 3 | 3 | 4 | 0 | 1 | 1 | 6 | 0 | 0 |
| COG0601 | ABC-type dipeptide/oligopeptide/nickel transport systems, permease components | 0 | 0 | 2 | 1 | 3 | 1 | 2 | 2 | 0 | 1 | 1 | 1 | 1 | 2 | 3 | 2 | 2 | 1 | 1 |
| COG0531 | Amino acid transporters | 0 | 3 | 0 | 0 | 2 | 2 | 2 | 0 | 1 | 4 | 2 | 3 | 1 | 0 | 0 | 1 | 0 | 0 | 3 |
| COG0444 | ABC-type dipeptide/oligopeptide/nickel transport system, ATPase component | 1 | 1 | 1 | 1 | 2 | 2 | 1 | 1 | 0 | 1 | 1 | 1 | 2 | 1 | 1 | 1 | 1 | 1 | 0 |
| COG4166 | ABC-type oligopeptide transport system, periplasmic component | 0 | 0 | 1 | 1 | 2 | 2 | 1 | 1 | 0 | 0 | 3 | 4 | 2 | 0 | 1 | 0 | 0 | 0 | 0 |
| COG3104 | Dipeptide/tripeptide permease | 0 | 1 | 0 | 1 | 1 | 1 | 2 | 1 | 1 | 2 | 1 | 1 | 2 | 1 | 1 | 1 | 0 | 0 | 1 |
| COG1173 | ABC-type dipeptide/oligopeptide/nickel transport systems, permease components | 0 | 0 | 1 | 1 | 2 | 1 | 1 | 2 | 0 | 1 | 1 | 1 | 1 | 1 | 1 | 1 | 1 | 0 | 1 |
| COG3842 | ABC-type spermidine/putrescine transport systems, ATPase components | 0 | 1 | 2 | 0 | 2 | 1 | 1 | 1 | 0 | 2 | 1 | 1 | 1 | 1 | 1 | 1 | 0 | 1 | 0 |
| COG0747 | ABC-type dipeptide transport system, periplasmic component | 0 | 1 | 1 | 1 | 1 | 2 | 1 | 1 | 0 | 1 | 0 | 0 | 0 | 1 | 1 | 1 | 2 | 1 | 0 |
| COG1280 | Putative threonine efflux protein | 0 | 1 | 0 | 1 | 0 | 0 | 0 | 1 | 1 | 1 | 2 | 2 | 1 | 1 | 1 | 1 | 0 | 0 | 1 |
| COG0410 | ABC-type branched-chain amino acid transport systems, ATPase component | 0 | 0 | 0 | 1 | 0 | 0 | 1 | 1 | 1 | 2 | 0 | 0 | 1 | 0 | 0 | 1 | 0 | 0 | 0 |
| COG0683 | ABC-type branched-chain amino acid transport systems, periplasmic component | 0 | 0 | 0 | 0 | 0 | 0 | 1 | 1 | 1 | 2 | 0 | 0 | 2 | 0 | 0 | 0 | 1 | 0 | 0 |
| COG0559 | Branched-chain amino acid ABC-type transport system, permease components | 0 | 0 | 0 | 1 | 0 | 0 | 1 | 1 | 1 | 2 | 0 | 0 | 1 | 0 | 0 | 1 | 0 | 0 | 0 |
| COG4177 | ABC-type branched-chain amino acid transport system, permease component | 0 | 0 | 0 | 1 | 0 | 0 | 1 | 0 | 1 | 2 | 0 | 0 | 1 | 0 | 0 | 1 | 0 | 0 | 0 |
| COG1177 | ABC-type spermidine/putrescine transport system, permease component II | 1 | 0 | 1 | 1 | 1 | 1 | 0 | 0 | 0 | 0 | 0 | 0 | 0 | 0 | 0 | 0 | 1 | 0 | 0 |
| COG0411 | ABC-type branched-chain amino acid transport systems, ATPase component | 0 | 0 | 0 | 0 | 0 | 0 | 1 | 1 | 1 | 1 | 0 | 0 | 1 | 0 | 0 | 0 | 0 | 0 | 0 |
| COG1176 | ABC-type spermidine/putrescine transport system, permease component I | 1 | 0 | 1 | 0 | 1 | 1 | 0 | 0 | 0 | 0 | 0 | 0 | 0 | 0 | 0 | 0 | 1 | 0 | 0 |
| COG0687 | Spermidine/putrescine-binding periplasmic protein | 1 | 0 | 1 | 1 | 1 | 0 | 0 | 0 | 0 | 0 | 0 | 0 | 0 | 0 | 0 | 0 | 1 | 0 | 0 |
| COG0687 | Spermidine/putrescine-binding periplasmic protein | 1 | 0 | 1 | 1 | 1 | 0 | 0 | 0 | 0 | 0 | 0 | 0 | 0 | 0 | 0 | 0 | 1 | 0 | 0 |
| COG0765 | ABC-type amino acid transport system, permease component | 0 | 1 | 0 | 0 | 1 | 1 | 1 | 0 | 0 | 0 | 0 | 0 | 0 | 0 | 0 | 0 | 0 | 0 | 0 |
| COG1126 | ABC-type polar amino acid transport system, ATPase component | 0 | 1 | 0 | 0 | 1 | 1 | 1 | 0 | 0 | 0 | 0 | 0 | 0 | 0 | 0 | 0 | 0 | 0 | 0 |
| COG0834 | ABC-type amino acid transport/signal transduction systems, periplasmic component | 0 | 1 | 0 | 0 | 1 | 0 | 1 | 0 | 0 | 0 | 0 | 0 | 0 | 0 | 0 | 0 | 0 | 0 | 0 |
| COG4608 | ABC-type oligopeptide transport system, ATPase component | 0 | 0 | 0 | 0 | 0 | 0 | 0 | 1 | 0 | 1 | 0 | 0 | 0 | 0 | 0 | 0 | 1 | 0 | 0 |
| COG4413 | Urea transporter | 0 | 1 | 0 | 0 | 0 | 0 | 0 | 1 | 0 | 1 | 0 | 0 | 0 | 0 | 0 | 0 | 0 | 0 | 0 |
| COG1115 | Na+/alanine symporter | 0 | 0 | 1 | 0 | 0 | 0 | 0 | 0 | 0 | 0 | 0 | 0 | 0 | 0 | 0 | 0 | 0 | 0 | 0 |
| **Sum** | | **7** | **12** | **15** | **14** | **23** | **18** | **21** | **18** | **12** | **29** | **15** | **17** | **21** | **8** | **11** | **13** | **18** | **4** | **7** |
