# Supplementary material for: Ecophysiology of Freshwater Verrucomicrobia Inferred from Metagenome-Assembled Genomes
Source: mSphere. 2017 Sep 27;2(5):e00277-17. doi: 10.1128/mSphere.00277-17 (PMC5615132; doi:10.1128/mSphere.00277-17)
Supplement: FIG S5 [file sph005172368sf6.pdf]

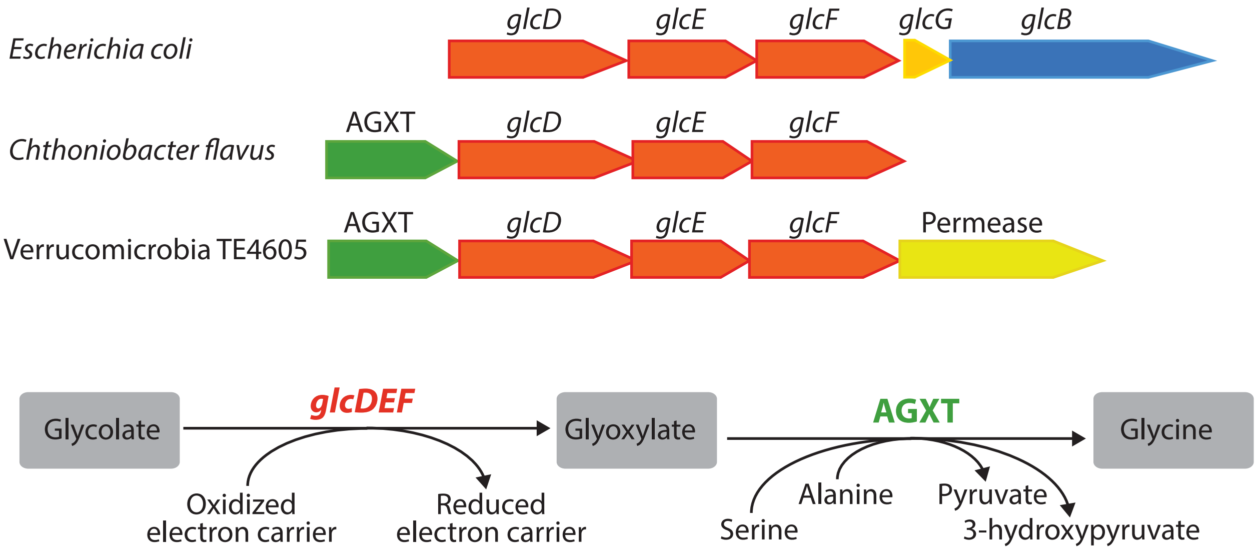

glcDEF: subuntis of glycolate oxidase

glcG: Uncharacterized *glc* operon protein GlcG

glcB: Malate synthase G

AGXT: Alanine-glyoxylate transaminase / serine-glyoxylate transaminase /serine-pyruvate transaminase

Permease: L-lactate permease family to which GlcA (glycolate permease) belongs to.
