# Supplementary material for: Ecophysiology of Freshwater Verrucomicrobia Inferred from Metagenome-Assembled Genomes
Source: mSphere. 2017 Sep 27;2(5):e00277-17. doi: 10.1128/mSphere.00277-17 (PMC5615132; doi:10.1128/mSphere.00277-17)
Supplement: FIG S6 [file sph005172368sf7.pdf]

Genes and Pathways	ME3880	TH2746	ME12612	ME12173	TE4605	ME6381	ME8366	TH2747	TH3004	TH0989	TH2519	TE1800	TH4590	ME2014	ME12657	TE1301	TH4093	ME30509	TH4820
**Central carbon metabolism**																			
Glycolysis (Embden-Meyerhof pathway), glucose => pyruvate	0.78	0.89	0.89	0.67	1	0.89	0.78	1	0.89	1	0.67	0.89	1	0.89	0.56	1	0.89	0.56	0.33
Glycolysis (Entner-Doudoroff pathway)	(0)	(0)	(0)	(0)	(0)	(0)	(0)	(0)	(0)	(0)	(0)	(0)	(0)	(0)	(0)	(0)	(0)	(0)	(0)
Pentose phosphate pathway (Pentose phosphate cycle)	0.86	0.71	0.71	0.71	0.86	0.57	0.71	1	0.86	1	0.71	0.86	1	0.86	0.57	1	0.71	0.29	0.43
Pyruvate oxidation, pyruvate => acetyl-CoA	1	1	1	0	1	1	1	1	1	1	1	1	1	1	1	1	1	1	1
Citrate cycle (TCA cycle, Krebs cycle)	0.63	0.88	1	0.38	0.75	0.63	0.88	0.88	0.88	0.75	1	1	0.88	0.63	0.75	1	0.5	0.25	0.25
**Other carbohydrate and carbon metabolism**																			
Galactose degradation to glycerate-3P	0.5	0.75	0.75	0.25	0.75	0.5	0.5	1	0.75	1	0.5	0.5	0.75	0.25	0.25	0.5	0.75	0.25	0.5
Rhamnose degradation	1	1	0.75	0.5	0.5	0.5	0.5	1	1	1	0.75	1	0.75	0.5	0.5	0.5	1	0.5	0.75
Fucose degradation	1	0.75	0.75	0.25	0.75	0.5	0.75	1	0.75	0.75	0.5	0.75	0.75	0.75	0.5	0.75	1	0.75	0.75
L-Arabinose degradation to xylulose-P for pentose pathway	1	1	0	0	0.67	0.33	0.33	1	1	1	0	0	0.33	0	0	0	0.67	0.33	0.33
Xylose degradation	1	1	1	1	1	0.5	1	1	1	1	0.5	1	1	1	1	1	0.5	1	1
D-Galacturonate degradation to pyruvate & D-glyceraldehyde 3P	(0)	(0)	(0)	(0)	0.8	0.2	(0)	0.6	(0)	(0)	(0)	(0)	(0)	(0)	(0)	(0)	(0)	(0)	(0)
D-Glucuronate degradation to pyruvate and D-glyceraldehyde 3P	0	0.6	0.4	0.4	0.6	0	0.2	0.6	0.8	0.6	0.6	0.8	0.6	0.2	0.4	0.4	0.6	0.4	0.8
Mannose degradation to glucose-P	0.5	1	1	1	1	1	1	1	1	1	1	1	1	1	0.5	0.5	1	0.5	1
Lactaldehyde degradation to pyruvate (Aerobic)	1	1	0.33	1	0.33	0.33	1	1	1	0.67	0.33	0.33	1	0.33	0.33	0.33	0.67	0.67	0.67
Fatty acid biosynthesis	1	1	1	1	1	1	1	1	1	1	1	1	1	1	1	1	1	1	1
Glycogen biosynthesis from alpha-D-glucose-6P via ADP-D glucose	0.75	0.25	0.75	0.75	0.75	0.5	1	1	0.75	1	0.5	0.5	1	1	1	1	0.5	0	0.75
Glyoxylate cycle	(0)	(0)	(0)	(0)	(0)	(0)	(0)	(0)	(0)	(0)	(0)	(0)	(0)	(0)	(0)	(0)	(0)	(0)	(0)
Ethylmalonyl pathway, alternative to glyoxylate cycle	0	0.43	0	0	0	0	0	0	0	0	0	0	0	0	0	0	0	0	0
**Hydrogenase**																			
Iron-only [FeFe]-hydrogenase	0	1	0	0	0	0	0	0	0	1	0	0	1	0	0	0	1	1	1
[NiFe]-hydrogenase																			
Group 1 [NiFe]-hydrogenase, H2 uptake	0	1	0	0	0	0	0	0	0	0	0	0	0	0	0	0	0	0	0
Group 2 [NiFe]-hydrogenase, H2 sensing	0	0	0	0	0	0	0	1	1	1	1	1	1	0	0	0	0	0	0
Group 3 [NiFe]-hydrogenase, NAD(P)-reducing	0	0	0	0	0	0	0	1	1	1	0	0	0	0	0	0	0	0	0
Group 4 [NiFe]-hydrogenase, H2 evolving	0	1	0	0	0	0	0	1	0	1	0	0	0.5	0	0	0	1	1	1
**Fermentation**																			
Pyruvate to acetate via acetyl-coA	1	1	0.67	0.67	0.33	0.33	1	1	1	0.67	0.33	0.33	1	0.67	0.67	0.67	1	1	1
Pyruvate to propanoate	(0)	(0)	(0)	(0)	(0)	(0)	(0)	(0)	(0)	(0)	(0)	(0)	(0)	(0)	(0)	(0)	(0)	(0)	(0)
Pyruvate to succinate	(0)	(0)	(0)	(0)	(0)	(0)	(0)	(0)	(0)	(0)	(0)	(0)	(0)	(0)	(0)	(0)	(0)	(0)	(0)
Pyruvate to butanoate	(0)	(0)	(0)	(0)	(0)	(0)	(0)	(0)	(0)	(0)	(0)	(0)	(0)	(0)	(0)	(0)	(0)	(0)	(0)
Pyruvate to butanol	0.71	0.71	0.71	0.29	1	0.71	0.57	0.43	0.43	0.43	0.57	0.57	1	0.57	0.57	0.57	0.43	0.43	0.43
Pyruvate to ethanol	(0)	(0)	(0)	(0)	(0)	(0)	(0)	(0)	(0)	(0)	(0)	(0)	(0)	(0)	(0)	(0)	(0)	(0)	(0)
Pyruvate to lactate	0	0	0	0	0	0	0	0	0	0	1	1	1	0	0	0	0	0	0
Pyruvate to acetone	(0)	(0)	(0)	(0)	(0)	(0)	(0)	(0)	(0)	(0)	(0)	(0)	(0)	(0)	(0)	(0)	(0)	(0)	(0)
**Nitrogen related**																			
Dissimilatory nitrate reduction, nitrate => ammonia	0	0	0	0	0	0	0	0	0	0	0	0	(0)	0	0	0	0	0	(0)
Denitrification, nitrate => nitrogen gas	0	0	0	0	0	0	0	0.25	0.25	0	0	0	0	0	0	0	0	0	0
TMAO (trimethylamine-N-oxide) reductioin	0	0	0	0	0	0	0	0	0	0	0	0	0	0	0	0	0	0	0
Nitrification, ammonia => nitrite	0	0	0	0	0	0	0	0	0	0	0	0	0	0	0	0	0	0	0
Nitrogen fixation, nitrogen => ammonia	0	0	0	0	0	0	0	1	0	1	0	0	0	0	0	0	1	0	0
Assimilatory nitrate reduction, nitrate => ammonia	0	1	0	0	(0)	0	0	0	0	0	0	0	0	0	0	0	(0)	0	(0)
Urease (Urea ==> CO2 + NH3)	0	0	0	0	0	0	0	0	0	1	0	0	1	0	0	1	0	0	0
Ammonia permease	1	1	1	1	1	1	1	1	1	1	0	0	1	0	1	1	1	0	1
**Sulfur related**																			
Dissimilatory sulfate reduction, sulfate => H2S	0	(0)	(0)	0	(0)	(0)	(0)	(0)	(0)	(0)	0	0	0.67	0	0	(0)	(0)	(0)	0
Dimethyl sulfoxide (DMSO) reduction	0	0	0	0	0	0	0	0	0	0	0	0	0	0	0	0	0	0	0
Polysulfide reduction	0	0	0	0	0	0	0	0	0	0	0	0	0	0	0	0	0	0	0
Thiosulfate oxidation by SOX complex, thiosulfate => sulfate	0	0	0	0	0	0	0	0	0	0	0	0	0	0	0	0	0	0	0
Alkanesulfonate oxidation to sulfite	0	0	0	0	0.5	0	0	0	0	0	0	0	0	0	0	0	0.5	0	0
ABC-type sulfate transport system	0	0.5	0	0	0.75	0	0	1	1	1	0.5	1	0	0	0	0	1	0	0.5
Sulfate permease	1	1	1	1	0	1	1	1	1	1	1	1	0	1	1	1	0	0	0
Assimilatory sulfate reduction, sulfate => H2S	0.67	0.67	1	0.67	1	0.67	1	0.67	1	1	0.33	0.33	1	0.33	1	1	0.67	1	0.33
**Phosphorus related**																			
Alkaline phosphatase	1	1	1	1	0	0	1	0	0	0	0	0	0	1	1	1	1	0	0
High-affinity phosphate specific transport (Pst) system	0	1	1	1	1	1	1	1	1	1	1	1	1	0	1	1	1	1	0
PiT inorganic phosphate transporter, low affinity	0	0	0	0	1	1	1	1	1	1	1	1	1	1	1	1	0	0	1
Polyphosphate storage and utilization	0	1	1	1	1	1	1	1	1	1	1	1	1	1	1	1	1	1	0
ABC-type phosphonate transport system	0	0	0	0	0	0	0	0	0	0	0	0	0	0	0	0	0	0	0
Methylphosphonate degradation	0	0	0	0	0	0	0	0	0	0	0	0	0	0	0	0	0	0	0
Phosphonoacetate degradation	0	1	0	1	1	0	1	1	1	0	0	0	1	0	0	0	0	0	0
**Sensitivity and tolorance to oxygen**																			
Superoxide dismutase (SOD)	1	1	1	1	1	1	1	1	1	1	1	1	1	1	1	1	0	0	1
Catalase-peroxidase, katG	0	1	0	1	0	0	0	0	0	1	0	0	1	1	1	1	0	0	0
**Oxidative phosphorylation**																			
NADH:quinone oxidoreductase (Complex I)	0.64	0.91	0.91	0.73	1	0.73	0.91	1	1	1	0.36	1	1	0.73	0.82	1	0.27	0.18	0.73
Succinate dehydrogenase complex (Complex II)	0.33	1	1	1	1	1	1	0.67	0.67	1	1	1	1	0.67	0	1	0	0	0
Cytochrome bc1 complex (Complex III)	0	0	0	0	0	0	0	0	0	0	0	0	0	0	0	0	0	0	0
Alternative Complex III, ACIII	0.33	1	0.5	0.83	1	0.33	1	1	1	1	0.67	1	1	1	0.83	1	0	0	0
Cytochrome c oxidase, aa3 type (Complex IV)	0	1	0.5	1	1	0.5	1	1	1	1	0	0	1	1	0.5	1	0	0	0
Cytochrome c oxidase, cbb3-type (Complex IV)	0	1	1	0.5	1	0	1	0.5	1	0.5	0.5	1	1	0.5	0.5	0.5	0	0	0
F-type ATPase complex (Complex V)	0	1	0.63	0	1	1	1	1	0.88	1	1	1	1	0.25	0	1	0	0	0
