# Supplementary material for: Ecophysiology of Freshwater Verrucomicrobia Inferred from Metagenome-Assembled Genomes
Source: mSphere. 2017 Sep 27;2(5):e00277-17. doi: 10.1128/mSphere.00277-17 (PMC5615132; doi:10.1128/mSphere.00277-17)
Supplement: FIG S7 [file sph005172368sf8.pdf]

pfam	Domain name	ME3880	TH2746	ME12612	ME12173	TE4605	ME6381	ME8366	TH2747	TH3004	TH0989	TH2519	TE1800	TH4590	ME2014	ME12657	TE1301	TH4093	ME30509	TH4820
pfam07635	PSCyt1	0	5	6	11	63	34	30	2	3	12	4	5	1	28	22	18	0	0	1
pfam07583	PSCyt2	1	5	6	12	50	29	26	2	2	12	1	2	1	22	17	14	0	0	3
pfam07587	PSD1	1	5	6	12	51	31	27	2	2	12	1	1	1	23	17	13	0	0	3
pfam07394	DUF1501	1	8	7	9	71	50	32	5	3	14	3	3	2	22	16	12	0	0	4
pfam07627	PSCyt3	0	0	2	4	17	6	6	0	0	1	1	1	0	9	3	5	0	0	0
pfam07624	PSD2	0	0	2	2	15	5	8	0	0	1	1	1	0	8	2	4	0	0	0
pfam07626	PSD3	0	0	2	3	16	6	2	0	0	1	1	1	0	7	2	4	0	0	0
pfam07631	PSD4	0	0	2	4	16	7	6	0	0	1	1	1	0	9	3	5	0	0	0
pfam07637	PSD5	0	0	2	4	16	6	2	0	0	1	1	1	0	9	4	5	0	0	0
pfam07586	DUF1552	0	0	2	1	17	6	6	0	0	1	1	1	0	9	4	5	0	0	0

(a)

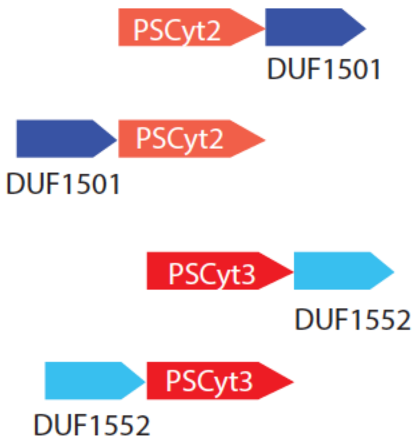

(b)
